# Tetrodotoxin for Chemotherapy-Induced Neuropathic Pain: A Randomized, Double-Blind, Placebo-Controlled, Parallel-Dose Finding Trial

**DOI:** 10.3390/toxins13040235

**Published:** 2021-03-25

**Authors:** Samuel A. Goldlust, Mojgan Kavoosi, Jennifer Nezzer, Mehran Kavoosi, Walter Korz, Kenneth Deck

**Affiliations:** 1 Hackensack University Medical Center, Hackensack, NJ 07601, USA; sgoldlust@hackensackUMC.org; 2 WEX Pharmaceuticals Inc., Vancouver, BC V6E 4A6, Canada; mehrank@wexpharma.com (M.K.); walterk@wexpharma.com (W.K.); 3 Premier Research Group, Morrisville, NC 27560, USA; jnezzer@araonline.net; 4 Alliance Research Centers, Laguna Hills, CA 92653, USA; kdeck@socsurgeons.com

**Keywords:** tetrodotoxin, pain, analgesic, voltage-gated sodium channels, clinical trial, chemotherapy induced neuropathic pain, peripheral neuropathy

## Abstract

Tetrodotoxin (TTX) has emerged as a potentially efficacious agent for chemotherapy-induced neuropathic pain (CINP), a prevalent, debilitating condition often resistant to analgesics. This randomized, double-blind, dose-finding study was undertaken to explore safety and trends in efficacy of four TTX doses and to identify a dose for further study. One hundred and twenty-five patients with taxane- or platinum-related CINP received subcutaneous placebo or TTX (7.5 µg twice daily (BID), 15 µg BID, 30 µg once daily (QD), 30 µg BID) for four consecutive days. Primary outcome measure was average patient-reported Numeric Pain Rating Scale (NPRS) score during Days 21–28 post-treatment. Changes in mean NPRS score were not statistically different between cohorts, due to small trial size and influence of a few robust placebo responders. Cumulative responder analysis showed significant difference from placebo with 30 µg BID cohort using the maximum response at any timepoint (*p* = 0.072), 5-day (*p* = 0.059), 10-day (*p* = 0.027), and 20-day (*p* = 0.071) rolling averages. In secondary quality of life (QOL) outcomes, 30 µg BID cohort also differed significantly from placebo in a number of SF-36 and CIPN20 subscales. Most adverse events (AE) were mild or moderate with oral paresthesia (29.6%) and oral hypoesthesia (24.8%) as most common.

## 1. Introduction

Chemotherapy-induced peripheral neuropathy (CIPN) develops in up to 80% of cancer patients receiving cytotoxic chemotherapeutics [1] and its severity and pathophysiology is dependent on the class of antineoplastic agent, dose and duration of treatment [2]. Neuropathic pain develops in up to 40% of patients [3,4,5] and often persists after termination of treatment. Chemotherapy-induced neuropathic pain (CINP) is a serious side effect that compromises a patient’s quality of life and leads to chemotherapy dose attenuation at the expense of survival [6,7,8,9].

A 2014 study by the American Society of Clinical Oncology reviewed 48 randomized clinical trials for CIPN [10]. The committee found no drugs to be efficacious at CIPN prevention. Only one drug, duloxetine, was recommended with ‘moderate confidence’ for treatment of existing CIPN, based upon a benefit versus placebo difference of less than 1 point on a 10-point scale on the Brief Pain Inventory-Short Form [11]. Moreover, there are no FDA-approved treatments for neuropathic pain resulting from chemotherapy, leaving a considerable unmet need.

The pathophysiology of CIPN is multifactorial. Neurotoxic chemotherapeutics alter ion channel and transient receptor potential (TRP) channel functioning, cause mitochondrial dysfunction and aberrant immune cell interactions, leading to erroneous somatosensory processing [12]. As the most prominent ion channel associated with CIPN, voltage-gated sodium channels (VGSCs) are integral to nociceptive signal propagation [13,14,15]. A clinical study found a causal relationship between polymorphisms in Na_v_1.4 and Na_v_1.8 genes and increased incidence and severity of oxaliplatin-induced neuropathy [16]. In animal models of neuropathic pain, Na_v_1.6 has been shown to contribute to both oxaliplatin-induced cold allodynia [17] and vincristine-induced mechanical allodynia [18]. Na_v_1.7 and Na_v_1.9 have also been implicated in oxaliplatin-induced hyperalgesia and cold allodynia [19,20] while increased Na_v_1.7 expression was observed in both human and rat dorsal root ganglion (DRG) following paclitaxel treatment [21,22]. Although the mechanisms leading to chemotherapy-induced changes in VGSC expression and function are not well understood, upregulated VGSC expression in primary afferent sensory neurons is known to be a common mechanism among three major classes of anti-neoplastic agents (taxanes, platinum compounds, vinca alkaloids) [2].

Tetrodotoxin (TTX), a highly specific VGSC blocker found in marine and terrestrial animals including pufferfish (genus Fugu), binds selectively to TTX-sensitive (TTX-IC_50_ ~10 nM) and TTX-resistant (TTX-IC_50_ ≥ 1 µM) VGSCs [23,24]. Tetrodotoxin binds to the extracellular vestibule of sodium channel, physically occluding the pore and thus, preventing sodium influx and action potential conduction in excitable cells [25]. Dysregulated VGSCs, namely TTX-sensitive Na_v_1.6 and Na_v_1.7 and TTX-resistant Na_v_1.8 and Na_v_1.9, in injured neurons and associated dorsal root ganglia (DRG) play a role in generating ectopic discharges associated with pain [26,27,28]. The analgesic properties of TTX are due in part to inhibition of these ectopic discharges [28,29]. Animal studies [29,30,31] and clinical trials in cancer–related pain [32,33,34,35] support that TTX is more potent than standard analgesics with durable benefit lasting weeks after last exposure [33].

This phase 2, randomized, double-blind, placebo-controlled, multicenter study of TTX for moderate to severe CINP was designed to assess the safety and tolerability of multiple doses and dosing intervals with the goal of identifying a dose for cohort expansion in further study. In addition to pre-planned primary and secondary analyses, several post hoc analyses were performed to more thoroughly investigate the efficacy and safety signals among cohorts.

## 2. Results

This study was not powered to detect statistical significance between cohorts. The analyses performed were intended to be descriptive, and to facilitate identification of the most promising dosage regimen for future studies. A total of 125 patients in the intent-to-treat (ITT) and 107 in the per-protocol (PP) populations were analyzed.

### 2.1. Patient Demographics

Between August 2012 and August 2014, a total of 125 patients at 24 U.S. sites were randomized, 121 (96.8%) completed the study, 2 (1.6%) were lost to follow-up, and 2 (1.6%) withdrew consent. Patient demographics and baseline characteristics (Table 1) were well matched between cohorts. The 125 patients enrolled had a mean age of 60.1 years (range 31–78) and stable mean (7-day average before randomization) baseline pain score of 6.5 ± 1.4 measured by numeric pain rating scale (NPRS).

### 2.2. Planned Outcome Measures

#### 2.2.1. Numeric Pain Rating Scale (NPRS) Score

For the primary outcome measure, the mean (±SD) change from baseline in average pain score for Days 22–28 was greatest in the TTX 30 µg QD cohort (−1.7 ± 2.3) followed by TTX 30 µg BID (−1.5 ± 1.8) (Table 2). Sensitivity analysis with the per-protocol population as well as with last observation carried forward (LOCF) and baseline observation carried forward (BOCF) in the intent-to-treat population supported the results of the primary outcome measure (WEX Pharmaceuticals Inc., data on file). The largest changes from baseline in average weekly pain scores during the 4-week trial were consistently observed in the TTX 30 µg QD and TTX 30 µg BID cohorts, although these changes did not reach statistical significance.

#### 2.2.2. SF-36, EORTC CIPN20, and PGIC

The Short Form Health (SF-36), European Organization for Research and Treatment of Cancer Chemotherapy-Induced Peripheral Neuropathy 20 (EORTC CIPN20) and Patient’s Global Impression of Change (PGIC) secondary outcome measurements for Day 28 are shown in Table 3. The weekly SF-36 and EORTC CIPN20 outcomes are shown in Figures S1 and S2, respectively. At Day 28, measurements trended towards the benefit of either TTX 15 µg BID or TTX 30 µg BID in all outcome subscales on the SF-36 and the sensory and motor nerve function subscales on the EORTC CIPN20. In addition, the TTX 30 µg BID cohort demonstrated statistically significant improvement compared to placebo on the SF-36 Body Pain (*p* = 0.004) and Physical Component (*p* = 0.076) subscales and the EORTC CIPN20 sensory symptom subscale (*p* = 0.091) at Day 28. A statistically significant improvement in sensory symptom scores is congruent with data indicating that TTX reduces heat hyperalgesia along with mechanical and cold allodynia in paclitaxel [36] and oxaliplatin treated mice [37]. Significant improvements in motor or autonomic function were not expected in this population. Patient’s overall impression of change as measured by the PGIC assessment at Day 28 indicated more patients in the TTX 30 µg BID cohort had the largest improvement after treatment with TTX. The complete PGIC assessment at Day 28 is shown in Table S1.

#### 2.2.3. Time to Peak Pain Relief and Use of Rescue Medication

The mean time to reach peak pain relief was about 3 weeks in all four TTX cohorts (Table 4). Despite the 3-week duration to reach peak relief, 20% of patients across all TTX treatment cohorts and 30.8% of patients in the TTX 30 µg BID cohort achieved a 30% reduction in pain severity during the first week of treatment (WEX Pharmaceuticals Inc., data on file). Moreover, the 30% reduction was maintained at Day 28 in 33% of patients across all treatment cohorts and by 38.5% of patients in the TTX 30 µg BID cohort (WEX Pharmaceuticals Inc., data on file), demonstrating a long duration effect in some patients. Additional post hoc analysis demonstrated most patients across all treatment cohorts did not require the use of extra rescue analgesics above baseline (WEX Pharmaceuticals Inc., data on file). The TTX 30 µg QD cohort demonstrated the longest time to first use of rescue medication and the shortest duration of usage of rescue medication (WEX Pharmaceuticals Inc., data on file).

### 2.3. Exploratory Outcome Measures

#### 2.3.1. Responder Analysis

A post hoc regression analysis of responders, considered patients with ≥30% improvement at any timepoint and in multi-day rolling pain score averages, revealed that patients in the TTX 30 µg BID cohort were more likely to be responders at any timepoint (Odds Ratio = 3.39, *p* = 0.072), as well as at the 5-day (Odds Ratio = 3.87, *p* = 0.059), 10-day (Odds Ratio = 3.90, *p* = 0.027), and 20-day rolling average timepoints (Odds Ratio = 2.90, *p* = 0.071) (Table 5).

#### 2.3.2. High Placebo Responders

**Figure 1:**
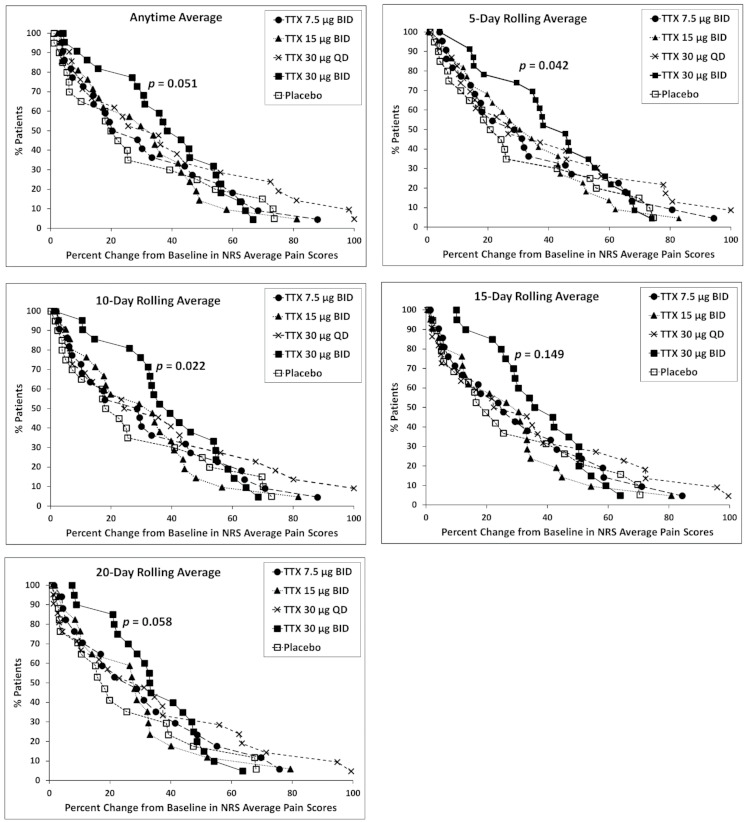
Cumulative responder analysis with all data from the single site removed. The *p*-value represents the significant difference from placebo of 30 µg BID (twice daily TTX) cohort using the maximum response of the cumulative averages.

A review of the placebo cohort showed a number of high placebo responders and a further inspection revealed a single site where both patients randomized to placebo cohort experienced dramatic pain relief (82% and 42% reduction in pain during Days 22–28). Post hoc analysis revealed that high placebo responders tended to show dramatic pain relief at multiple timepoints. Thus, if these high placebo responders can be identified in advance, separation of the groups should improve across all time periods. The analysis also highlighted that even a small number of high placebo responders can significantly influence outcomes. When the study was re-evaluated with all data from this single site removed, the same trends in the primary and secondary outcome measures were observed but considerably closer to statistical significance with many more cases reaching *p* < 0.05 and *p* < 0.01 (Figures S1–S3). The cumulative responder analysis (Figure 1) demonstrates the TTX 30 µg BID cohort continued to show the largest number of patients with ≥30% improvement from baseline in average pain scores at any timepoint (*p* = 0.051), 5-day (*p* = 0.042), 10-day (*p* = 0.022), 15-day (*p* = 0.149), and 20-day (*p* = 0.058) rolling averages.

### 2.4. Safety and Tolerability

Adverse events (AE) are summarized in Table 6 with the complete AE profile shown in Table S2. Across the TTX cohorts, 80.0 to 92.3% of patients experienced at least one AE. The most frequent AEs were oral paresthesia (34/100 TTX patients, 3/25 placebo) and oral hypoesthesia (28/100 TTX patients, 3/25 placebo). The median onset and duration of all mild and moderate AEs deemed related to TTX displayed a combined average of 1 h:16 min and 16 h:12 min, respectively. Table 7 shows the onset and duration of events related to hypoesthesia and paresthesia for all cohorts. All AEs were mild or moderate with the exception of 4 patients with severe AEs in the TTX 30 µg BID group, 3 of whom had events which were deemed related or possibly related to treatment (hypertension, paresthesia, extremity pain, burning sensation), and one patient whose death from metastatic carcinoma was deemed unrelated to TTX. Two patients withdrew from the study due to AEs: one patient in the 15 µg BID cohort (vertigo, moderate, related) and one patient in the 30 µg BID cohort (vertigo and influenza-like illness, both moderate, both possibly related). In the TTX 30 µg QD cohort, two patients with pre-existing history of hypertension experienced mild and severe hypertension that was deemed as possibly related to TTX and both resolved without further treatment. Another patient in the TTX 30 µg QD cohort experienced mild QT prolongation that was unchanged from baseline (Table S2). A study completed in healthy adults has demonstrated that exposure up to TTX 45 µg QD (corresponding to higher TTX plasma blood C_max_ than 30 µg BID) does not cause blood pressure or ECG changes as determined by concentration-QTc analysis [38].

## 3. Discussion

In clinical practice, a variety of drugs are used to treat CINP, including anticonvulsants, opiates, antidepressants, and NSAIDs. The potential for side effects due to off target effects (e.g., constipation, fatigue, renal dysfunction) in a susceptible population and scant supporting evidence highlight the need for clinical trials of novel therapies. This unmet need has similarly been recognized by the National Cancer Institute, and a chemotherapy induced peripheral neuropathy working group has been established [39].

Voltage-gated sodium channels (VGSCs) have been shown to underlie nociceptive signaling with increased gating properties correlated with increased pain sensation [15,40]. TTX is a specific blocker of VGSCs with high affinity for TTX-sensitive VGSCs found in peripheral nerves. Like other toxins used in medicine (e.g., botulinum toxin type A, morphine, curare), therapeutic doses of TTX are safe and well-tolerated [38]. As a peripherally acting, non-addictive analgesic targeting TTX-sensitive VGSCs, TTX is a rational candidate for patients with inadequately controlled CINP.

This exploratory, dose-finding study was powered to detect trends, not significant differences between cohorts. Both planned and post hoc exploratory analyses were performed to facilitate identification of a dose for further study. The TTX 30 µg QD and TTX 30 µg BID cohorts demonstrated the greatest change from baseline in average weekly pain scores at all weekly timepoints. Both sensitivity using per-protocol population and LOCF/BOCF analyses supported this observation. Based upon recommendations from the Initiative on Methods, Measurement, and Pain Assessment in Clinical Trials (IMMPACT), a 30% decrease in pain severity is considered a moderately important and clinically meaningful improvement [41,42]. Among all cohorts, TTX 30 µg BID consistently led to the greatest number of responders experiencing at least a 30% reduction in pain intensity at any time point and in multi-day rolling averages.

IMMPACT recommendations also include the use of assessments to measure physical and emotional functioning and global impression of improvement, as part of a comprehensive evaluation of treatment outcome in placebo-controlled clinical trials for chronic pain [43]. In this study, three quality of life assessments (SF-36, EORTC CIPN 20, PGIC) were used to ensure a broad assessment. The SF-36 questionnaire assessed the patient’s emotional state and ability to perform everyday activities. The EORTC CIPN 20 was used to assess sensory, motor, and autonomic symptoms [44]. Lastly, the PGIC evaluated quality of life, physical condition, emotional state, social life, and change in neuropathy. Although both the TTX 15 µg BID and TTX 30 µg BID cohorts demonstrated improvements in quality of life compared to placebo, TTX 30 µg BID consistently led to the greatest improvement from baseline in all three assessments at Day 28. TTX has been shown to reduce paclitaxel-induced heat hyperalgesia as well as mechanical and cold allodynia in mice [36,37] which is congruent with the significant improvement in the SF-35 Physical Component and Body Pain subscales and the EORTC CIPN 20 Sensory subscale observed with TTX 30 µg BID at Day 28.

In addition, 38.5% and 33% of patients in the TTX 30 µg BID and overall TTX treatment cohort, respectively, reported at least a 30% reduction in pain during week 4 post-treatment. This pain relief observed beyond week 1 post-treatment occurred in the absence of any residual systemic TTX. Prior pharmacokinetic analysis has revealed TTX has a t_1/2_ of approximately 4.5 h and is almost exclusively eliminated (approximately 96.75% of dose) within 36 h [38]. The absence beyond week 1 of any AEs associated with TTX such as hypoesthesia, oral hypoesthesia, paresthesia, and oral paresthesia also support the lack of residual systemic TTX as the cause of the observed long-duration pain relief. Long-acting analgesia following TTX administration has also been observed in a phase III cancer-related pain trial where patients receiving TTX 30 µg BID for four day were found to have a mean duration of analgesia of 56.7 days compared to 9.9 days for placebo [33]. It is postulated that TTX temporarily inhibits the repetitive ectopic discharges from damaged peripheral neurons, thereby disrupting the pain-dependent synaptic plasticity of the spinal cord and brain. This disruption reduces or eliminates the existing pain hypersensitivity and increases the pain threshold, resulting in the observed long-duration pain relief [45]. The occurrence of pain-dependent central sensitization in the spinal cord and brain and the resulting post-injury pain hypersensitivity is well documented [45]. Neuropathic pain patients taking other sodium channel blockers have also reported pain relief lasting days and weeks after their last treatment [40]. Thus, it is believed that some CINP patients may experience a prolonged benefit lasting weeks to months after the last dose of TTX. A similar phenomenon has been observed in rats with peripheral neuropathy. Intravenous administration of lidocaine has been shown to eliminate mechanical allodynia for up to 3 weeks [46] and a single injection of bupivacaine to block the sciatic nerve in rats was observed to relieve heat hyperalgesia for at least 2 days [47].

Double-blind, randomized, placebo-controlled trials are the gold standard in evaluating novel therapeutics. However, in trials with subjective outcomes such as pain or quality of life, the expectation of therapeutic benefit often leads to a placebo response [48]. In fact, analgesic trials are challenged by a high rate of placebo responders [49]. A meta-analysis of 215 randomized clinical trials of patients with osteoarthritis pain revealed the placebo response can be as high as 75% [50]. The doctor-patient relationship and the healthcare setting were shown to be important contributors to this effect [51,52]. Given the small size of the placebo cohort in the present study, the high placebo responders exerted a considerable influence on study outcomes. Post hoc analyses revealed that removal of only two robust placebo responders helped to support identification of a TTX regimen meriting further study. Future well powered trials will serve to mitigate this placebo effect.

TTX was generally well tolerated despite the many concomitant medications taken by the patients in this study. The most frequent AEs in the TTX treatment cohorts were paresthesia, oral paresthesia, oral hypoesthesia, headache, and dizziness. As known properties of TTX, paresthesia and hypoesthesia are generally mild to moderate and transient as observed in the present study. There were three reported serious AEs, two due to underlying metastatic disease and one due to severe viral upper respiratory infection; all were deemed unrelated to TTX and did not lead to termination of study treatment. However, two patients did withdraw consent due to AEs, and both recovered without issue.

## 4. Conclusions

This randomized, double blind, placebo controlled, parallel dose comparison provides direction for further study of TTX. Pre-planned and post hoc analyses suggest an analgesic signal at TTX 30 µg BID dosed for 4 days every 3 weeks, and safety data support a favorable risk/benefit profile in a population with limited therapeutic options. Development of a sufficiently powered study at this dose and schedule is underway.

## 5. Materials and Methods

### 5.1. Standard Protocol Approvals, Registrations, and Patient Consents

The trial was conducted at 24 clinical sites across the USA and in accordance with the ethical principles originating from the Declaration of Helsinki and current Good Clinical Practices and in compliance with local regulatory requirements and 21 CFR 312. The study protocol was approved by the local institutional review board or independent ethics committee at each participating site. Informed consent was obtained from all patients prior to screening. The study was registered at ClinicalTrials.gov (Identifier NCT01655823).

### 5.2. Participants

To qualify for the study, patients had to be ≥18 years of age, have taxane- or platinum-based CINP for least 1 month and no evidence of progressive cancer. Other key inclusion criteria included: no significant residual chemotherapy-induced toxicity other than neuropathic pain, a Douleur Neuropathique 4 questionnaire score ≥4, an average daily Numeric Pain Rating Scale (NPRS) score ≥4 with a fluctuation ≤ ±2 for one week, and an Eastern Cooperative Oncology Group performance status score of 0 or 1. Patients on stable doses of anticonvulsants and anti-depressants were included.

Patients who met any of the following criteria were excluded: neuropathic pain not caused by chemotherapy, current use of drugs known to cause neuropathic pain, current use of other therapy for treatment of neuropathic pain, use of controlled-release opioids within 7 days of baseline period or the expectation to use controlled-release opioids, screening serum creatinine >1.5× normal, bone metastases, predicted life expectancy <8 months, current use of lidocaine or other antiarrhythmics, current use of scopolamine and acetylcholinesterase-inhibitors, and CINP attributed to bortezomib, vinca alkaloids, or analogues.

### 5.3. Study Design

**Figure 2:**
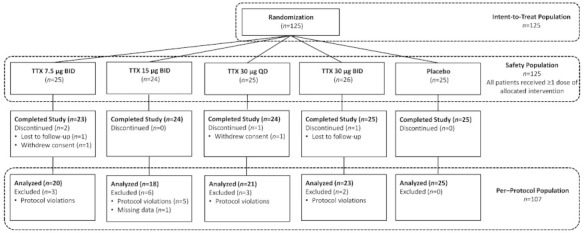
Participant disposition and trial profile.

One hundred and twenty-five patients with moderate to severe CINP were randomized. Patients provided informed consent and underwent screening within 30 days of randomization; 7 days prior to randomization, patients began a Baseline Period to establish stable pain and other eligibility criteria. On Day 1, patients were randomized in a 1:1:1:1:1 ratio to one of five cohorts: placebo BID, TTX 7.5 µg BID, TTX 15 µg BID, TTX 30 µg QD, or TTX 30 µg BID (Figure 2). Patients received subcutaneous injections in the thigh or abdomen twice daily for 4 consecutive days (Days 1–4). To maintain blinding, the second daily injection for patients in the TTX 30 µg QD cohort was TTX-matched placebo. Both TTX (Halneuron^®^) and TTX-matched placebo were manufactured by K.A.B.S. Laboratories Inc (Saint Hubert, QC, Canada) for WEX Pharmaceuticals Inc. (Vancouver, BC, Canada). Patients were evaluated daily during the 4-day treatment period and on Days 7, 14, 21, and 28 post-treatment for safety and efficacy.

### 5.4. Randomization and Blinding

The contract research organization (Premier Research Group, Morrisville, NC, USA) generated the randomization schedule stratified by gender. A block size of five for a total of 60 blocks were prepared with 30 blocks per gender. The random allocation sequence was generated using a permuted block procedure using a random seed. The randomization schedule was inputted into a central Interactive Web Response System (IWRS) to maintain the double-blind process across multiple sites. IWRS performed the assignment of patients to each treatment arm and presented the assignment only to one designated pharmacist at each site in attempt to safeguard the integrity of the binding. All other study personnel and patients remained blinded. All data were stored in a central data repository and transferred for analysis following database lock.

### 5.5. Outcome Measures

The primary efficacy outcome was a change from baseline in weekly average Numeric Pain Rating Scale (NPRS) score at 22–28 days post-treatment. NPRS scores were collected via an interactive web/voice response phone system three times daily until end of study (Day 28). Secondary efficacy endpoints included time to peak pain relief, use of rescue analgesia, scores on the Short Form Health (SF-36) and European Organization for Research and Treatment of Cancer Chemotherapy-Induced Peripheral Neuropathy 20 (EORTC CIPN20) questionnaires (both collected at baseline and Days 7, 14, 21, and 28) and the Patient’s Global Impression of Change (PGIC) questionnaire completed on Day 28. Safety endpoints included: incidence of adverse events (AE) and serious adverse events (SAE), physical examination findings, changes from baseline in vital signs, laboratory (hematology, chemistry, and urinalysis) results, and ECG assessments. The Medical Dictionary for Regulatory Activities was used to classify all AE by system organ class and preferred term. The investigator assessed whether each AE was “not related”, “unlikely related”, “possibly related” or “related” to TTX.

### 5.6. Safety Monitoring

Patients were clinically evaluated daily during the 4-day treatment period, as well as Days 7, 14, 21, and 28. Patients experiencing an AE related to the study medication were monitored until resolution or clinical judgment indicated that no further evaluation was warranted. Per protocol, a data safety monitoring committee oversaw the safety population (considered any patient who received study medication).

### 5.7. Statistical Analysis

All analyses were intended to be descriptive and the application of a significance level of *p* < 0.1 was intended to assist in the identification of the most promising dosing regimen for further study. Continuous outcome measures including change in daily and weekly average NPRS scores were assessed using restricted maximum likelihood-based mixed-model repeated-measures analysis. The model included treatment, study day (or week), treatment-by-day (or week) interaction, and baseline score as covariates. All statistical analyses were performed on the intention-to-treat (ITT) population (except a per-protocol [PP] sensitivity analysis performed for the primary efficacy outcome). Other sensitivity analyses were also performed for the primary outcome to investigate model assumptions.

Responder rates for patients who achieve a reduction in pain evaluated at a continuous cut-off ranging from 0% to 100% reduction in weekly average NPRS scores from Baseline were first determined and then, proportion of subjects who showed 30% or better response were analyzed using a logistic regression model with treatment, gender and baseline score as covariates and the odds ratios, 95% CIs, and *p*-values were presented for each visit. For efficacy outcomes that were ordinal or binary/nominal, nominal *p* values from a Cochran-Mantel-Haenszel test [53,54] or from Fisher’s exact test [55] were calculated.

## Figures and Tables

**Table 1:** Demographics and baseline characteristics.

	TTX Dosage				
Variable	7.5 µg BID (N = 25)	15 µg BID (N = 24)	30 µg QD (N = 25)	30 µg BID (N = 26)	Placebo (N = 25)
Age, mean (±SD), y	59.1 (±10.3)	61.4 (±10.0)	60.4 (±10.3)	60.6 (±11.1)	59.0 (±10.3)
Female, *n* (%)	16 (64.0)	15 (62.5)	15 (60.0)	16 (61.5)	15 (60.0)
BMI, mean (±SD), kg m^−2^	30.6 (±6.4)	28.6 (±6.5)	30.1 (±7.8)	30.1 (±7.9)	32.6 (±10.0)
Baseline NPRS, mean ±SD	6.3 (±1.3)	7.0 (±1.4)	6.2 (±1.2)	6.3 (±1.4)	6.7 (±1.5)
CINP Duration, Mean (±SD), y					
Lower extremities	3.4 (±5.4)	2.1 (±2.3)	1.7 (±2.1)	2.4 (±1.9)	3.1 (±3.1)
Upper extremities	3.5 (±5.6)	1.8 (±1.8)	1.9 (±2.2)	2.4 (±2.0)	2.7 (±2.9)
Prior Medications					
Opiates, *n* (%) ^a^	7 (28.0)	7 (29.2)	8 (32.0)	6 (23.1)	9 (29.6)
SNRI, *n* (%)	2 (8.0)	2 (8.3)	5 (20.0)	4 (15.4)	4 (16.0)
Anticonvulsants, n (%) ^b^	9 (36.0)	8 (33.3)	4 (16.0)	10 (38.5)	4 (16.0)
NSAIDs, *n* (%) ^c^	5 (20.0)	7 (29.2)	2 (8.0)	3 (11.5)	5 (20.0)
Prior radiation, *n* (%)	13 (52.0)	10 (41.7)	13 (52.0)	14 (53.8)	11 (44.0)
Primary Cancer, *n* (%)					
Colon	10 (40.0)	7 (29.2)	7 (28.0)	8 (30.8)	12 (48.0)
Breast	9 (36.0)	6 (25.0)	9 (36.0)	10 (38.5)	5 (20.0)
Other	6 (24.0)	11 (45.8)	9 (36.0)	8 (30.8)	8 (32.0)
Chemotherapy Considered to Cause Pain, *n* ^d^					
Taxane	7	10	12	11	6
Platinum	19	16	15	16	19

BMI: body mass index; CINP: chemotherapy induced neuropathic pain; NPRS: Numerical Pain Rating Scale; NSAIDs: non-steroidal anti-inflammatory drugs; SNRI: selective norepinephrine reuptake inhibitor; TTX: tetrodotoxin; BID: twice daily; QD: once daily; *n*: number of subjects. ^a^ Natural opium alkaloids. ^b^ Gabapentin, pregabalin, topiramate. ^c^ Propionic acid derivatives. ^d^ Taxane and platinum were both counted in some patients.

**Table 2:** Change from baseline in weekly average pain scores.

	TTX Dosage				
Time Period	7.5 µg BID (N = 25)	15 µg BID (N = 24)	30 µg QD (N = 25)	30 µg BID (N = 26)	Placebo (N = 25)
Days 1–7, *n*	25	24	24	25	25
Mean change in NPRS (±SD)	−0.8 (±1.0)	−0.9 (±0.8)	−1.0 (±1.6)	−1.2 (±1.6)	−0.9 (±1.1)
*p*-value for Difference in LSM vs. Placebo	0.84	0.84	0.66	0.35	
Days 8–14, *n*	23	24	24	25	24
Mean change in NPRS (±SD)	−1.2 (±1.4)	−1.2 (±1.1)	−1.5 (±1.8)	−1.4 (±1.8)	−1.4 (±1.7)
*p*-value for Difference in LSM vs. Placebo	0.57	0.61	0.69	0.94	
Days 15–21, *n*	22	22	23	23	25
Mean change in NPRS (±SD)	−1.2 (±1.5)	−1.3 (±1.6)	−1.7 (±2.0)	−1.6 (±1.6)	−1.4 (±1.9)
*p*-value for Difference in LSM vs. Placebo	0.90	0.85	0.58	0.90	
Days 22–28, *n*	21	21	23	24	25
Mean change in NPRS (±SD)	−1.3 (±1.4)	−1.1 (±1.6)	−1.7 (±2.3)	−1.5 (±1.8)	−1.3 (±2.1)
*p*-value for Difference in LSM vs. Placebo	1.0	0.68	0.58	0.92	

NPRS: Numerical Pain Rating Scale; LSM: least squares mean; SD: standard deviation; TTX: tetrodotoxin; BID: twice daily; QD: once daily; *n*: number of subjects. A comparison of least squares mean differences (treatment—placebo) using a mixed-effect model repeated measure model (with treatment as the main effect, gender, baseline NPRS score, week and treatment-by-week interaction as covariates) found no statistically significant differences.

**Table 3:** Change from baseline in Short Form 36 and European Organization for Research and Treatment of Cancer (EORTC) CIPN20 outcome measures at Day 28 post-treatment.

	TTX Dosage				
	7.5 µg BID N = 25	15 µg BID N = 24	30 µg QD N = 25	30 µg BID N = 26	Placebo N = 25
SF-36 Mental Component Score					
*n*	23	24	24	24	24
Mean change (±SD)	−0.23 (±8.8)	4.1 (±8.0)	−0.08 (±9.1)	2.6 (±9.3)	0.03 (±7.6)
Difference in LSM vs. Placebo (95% CI)	0.5 (−3.9, 4.9)	3.6 (−0.8, 8.0)	−1.2 (−5.6, 3.2)	2.7 (−1.7, 7.1)	
SF-36 Physical Component Score					
*n*	23	24	24	24	24
Mean change (±SD)	2.1 (±8.7)	4.2 (±7.6)	2.3 (±5.3)	6.8 (±7.3)	3.1 (±7.2)
Difference in LSM vs. Placebo (95% CI)	−1.5 (−5.4, 2.4)	0.37 (−3.5, 4.3)	−1.0 (−4.9, 2.8)	3.5 (−0.4, 7.4) ^a^	
SF-36 Mental Health Score					
*n*	23	24	24	24	24
Mean change (±SD)	0.22 (±9.8)	3.2 (±10.6)	−0.6 (±8.2)	3.5 (±10.2)	0.5 (±5.8)
Difference in LSM vs. Placebo (95% CI)	0.4 (−4.3, 5.1)	2.7 (−2.0, 7.3)	−1.6 (−6.3, 3.0)	3.1 (−1.5, 7.7)	
SF-36 Role Emotional Score					
*n*	23	24	24	25	24
Mean change (±SD)	0.66 (±13.4)	6.8 (±10.1)	2.2 (±9.5)	2.4 (±13.1)	0.16 (±11.9)
Difference in LSM vs. Placebo (95% CI)	1.2 (−4.7, 7.0)	4.2 (−1.6, 10.0)	−0.57 (−6.4, 5.2)	2.5 (−3.3, 8.2)	
SF-36 Social Functioning Score					
*n*	23	24	24	25	24
Mean change (±SD)	1.7 (±10.3)	4.7 (±11.6)	0.41 (±8.1)	5.5 (±8.6)	1.0 (±7.1)
Difference in LSM vs. Placebo (95% CI)	−0.35 (−5.1, 4.4)	2.6 (−2.1, 7.3)	−1.2 (−5.8, 3.5)	4.4 (−0.4, 9.0)	
SF-36 Vitality/Energy Score					
*n*	23	24	24	24	24
Mean change (±SD)	−0.95 (±10.7)	2.6 (±9.2)	1.3 (±10.1)	5.0 (±8.7)	3.1 (±6.2)
Difference in LSM vs. Placebo (95% CI)	−3.2 (−7.9, 1.6)	−0.55 (−5.2, 4.2)	−2.3 (−7.6, 1.8)	2.1 (−2.6, 6.8)	
SF-36 General Health Score					
*n*	23	24	24	25	25
Mean change (±SD)	0.04 (±6.3)	3.1 (±8.9)	−0.90 (±5.6)	1.5 (±6.1)	−0.25 (±6.6)
Difference in LSM vs. Placebo (95% CI)	0.15 (−3.4, 3.7)	3.4 (−0.2, 7.0)	−0.88 (−4.4, 2.7)	2.3 (−1.3, 5.8)	
SF-36 Body Pain Score					
*n*	23	24	24	25	24
Mean (±SD)	3.6 (±8.0)	3.7 (±8.3)	2.9 (±6.7)	9.2 (±7.1)	2.6 (±8.3)
Difference in LSM vs. Placebo (95% CI)	0.11 (−4.0, 4.2)	−0.08 (−4.2, 4.0)	−0.07 (−4.1, 4.0)	6.0 (2.0, 10.0) ^b^	
SF-36 Role Physical Score					
*n*	23	24	24	25	24
Mean (±SD)	2.1 (±9.4)	6.6 (±13.2)	2.1 (±7.4)	6.9 (±9.7)	3.3 (±8.0)
Difference in LSM vs. Placebo (95% CI)	−0.98 (−6.0, 4.0)	1.2 (−3.8, 6.3)	−2.0 (−7.0, 3.0)	3.6 (−1.3, 8.5)	
SF-36 Physical Functioning Score					
*n*	23	24	24	25	25
Mean (±SD)	1.1 (±10.1)	4.6 (±7.5)	2.7 (±5.2)	4.6 (±7.8)	2.6 (±6.6)
Difference in LSM vs. Placebo (95% CI)	−2.1 (−6.1, 1.9)	1.2 (−2.9, 5.2)	−0.80 (−4.8, 3.2)	1.9 (−2.1, 5.9)	
CIPN20 Sensory Scale					
*n*	23	24	23	24	25
Mean (±SD)	−4.52 (±5.9)	−5.17 (±4.7)	−4.61 (±5.0)	−4.6 (±4.6)	−2.2 (±5.0)
Difference in LSM vs. Placebo (95% CI)	−1.7 (−4.3, 0.93)	−1.5 (−4.2, 1.2)	−1.6 (−4.3, 1.0)	−2.2 (−4.8, 0.37) ^c^	
CIPN20 Motor Scale					
*n*	23	24	23	24	25
Mean (±SD)	−2.2 (±4.8)	−2.4 (±4.1)	−2.1 (±3.8)	−2.8 (±3.3)	−2.0 (±3.2)
Difference in LSM vs. Placebo (95% CI)	0.53 (−1.4, 2.4)	0.63 (−1.3, 2.5)	0.58 (−1.3, 2.5)	−0.63 (−2.5, 1.3)	
CIPN20 Autonomic Scale					
*n*	23	24	23	24	25
Mean (±SD)	−0.83 (±1.6)	−0.33 (±1.4)	−0.74 (±1.5)	−0.33 (±0.6)	−0.08 (±1.2)
Difference in LSM vs. Placebo (95% CI)	−0.40 (−1.0, 0.2)	0.08 (−0.5, 0.7)	−0.28 (−0.9, 0.3)	−0.27 (−0.9, 0.3)	
PGIC—Overall QoL					
Most Frequent Reported Impression of Change	About the same	About the same	About the same	Moderately better	About the same
*n* (%)	11 (44.0%)	8 (33.3%)	9 (36.0%)	10 (38.5%)	9 (36.0%)
PGIC—Physical Condition					
Most Frequent Reported Impression of Change	About the same	About the same;A little better	About the same	Moderately better	About the same
*n* (%)	13 (52.0%)	8 (33.3%) 8 (33.3%)	10 (40.0%)	9 (34.6%)	11 (44.0%)
PGIC—Emotional State					
Most Frequent Reported Impression of Change	About the same	About the same	About the same	About the same	About the same
*n* (%)	17 (68.0%)	15 (62.5%)	14 (56.0%)	12 (46.2%)	11 (44.0%)
PGIC—Enjoy Social Life					
Most Frequent Reported Impression of Change	About the same	About the same	About the same	About the same	About the same
*n* (%)	16 (64.0%)	15 (62.5%)	15 (60.0%)	12 (46.2%)	12 (48.0%)
PGIC—Numbness, Tingling or Pain in Hands or Feet					
Most Frequent Reported Impression of Change	About the same	About the same	About the same	About the same;Moderately better	About the same
*n* (%)	10 (40.0%)	8 (33.3%)	8 (32.0%)	6 (23.1%) 6 (23.1%)	9 (36.0%)
PGIC—Believe Receiving Active Agent					
Yes, *n* (%)	15 (60.0%)	15 (62.5%)	14 (56.0%)	22 (84.6%)	13 (52.0%)
No, *n* (%)	8 (32.0%)	7 (37.5%)	8 (32.0%)	3 (11.5%)	12 (48.0%)

CI: confidence interval; CIPN20: European Organization for Research and Treatment of Cancer Chemotherapy-Induced Peripheral Neuropathy 20; LSM: least squares mean; SD: standard deviation; SF-36: Medical Outcomes Study Short Form Survey; TTX: tetrodotoxin; QoL: quality of life; BID: twice daily; QD: once daily; *n*: number of subjects. Least squares mean differences (treatment—placebo) used a mixed-effect model repeated measure model (with treatment as the main effect, gender, baseline NPRS score, week and treatment-by-week interaction as covariates). An unstructured covariance matrix was used to model the within-patient correlation. ^a^ Statistically significant at *p* = 0.076. ^b^ Statistically significant at *p* = 0.004. ^c^ Statistically significant at *p* = 0.091.

**Table 4:** Time to peak pain relief.

	TTX Dosage				
	7.5 µg BID N = 25	15 µg BID N = 24	30 µg QD N = 25	30 µg BID N = 26	Placebo N = 25
Time to Peak Pain Relief ^a^					
*n*	23	24	23	25	25
Mean change (Weeks) (±SD)	2.5 (±1.2)	2.4 (±1.2)	3.0 (±1.2)	2.6 (±1.3)	2.5 (±1.1)
Kaplan–Meier Estimate of weeks to peak pain relief					
Median (Weeks) (95% CI)	3.00 (1.00, 3.00)	2.50 (1.00, 3.00)	3.00 (3.00, 4.00)	3.00 (1.00, 4.00)	3.00 (1.00, 3.00)

CI: confidence interval; NPRS: Numerical Pain Rating Scale; SD: standard deviation; TTX: tetrodotoxin; BID: twice daily; QD: once daily; *n*: number of subjects. Note: Time to peak pain relief defined as the time from first dose of study medication to the date of the maximum pain relief. Week 1 = Days 1 to 7, Week 2 = Days 8 to 14, Week 3 = Days 15 to 21, Week 4 = Days 22 to 28. ^a^ Subjects who withdrew from the study early or subjects who completed the study but took rescue on their last visit were censored at their last visit with the last non-missing average pain score.

**Table 5:** Responder analysis (≥30% improvement in pain score).

	TTX Dosage				
Responders	7.5 µg BID (*n* = 25)	15 µg BID (*n* = 24)	30 µg QD (*n* = 25)	30 µg BID (*n* = 26)	Placebo (*n* = 25)
Any timepoint, *n* (%) ^a^	9 (36.0%)	11 (45.8%)	10 (40.0%)	15 (57.7%)	8 (32.0%)
Odds ratio vs. placebo	1.12	1.99	1.65	3.39 ^a^	
(95% CI)	(0.32, 3.96)	(0.56, 7.13)	(0.46, 5.90)	(0.96, 11.97)	
5-day rolling averages, *n* (%) ^b^	10 (40.0%)	12 (50.0%)	10 (40.0%)	16 (61.5%)	8 (32.0%)
Odds ratio vs. placebo	1.33	2.54	1.58	3.87 ^b^	
(95% CI)	(0.38, 4.65)	(0.71, 9.06)	(0.45, 5.62)	(1.10, 13.61)	
10-day rolling averages, *n* (%) ^c^	8 (32.0%)	10 (41.7%)	10 (40.0%)	15 (57.7%)	8 (32.0%)
Odds ratio vs. placebo	0.90	1.68	1.63	3.90 ^c^	
(95% CI)	(0.25, 3.24)	(0.47, 6.06)	(0.45, 5.83)	(1.08, 14.09)	
15-day rolling averages, *n* (%)	8 (32.0%)	10 (41.7%)	10 (40.0%)	12 (46.2%)	8 (32.0%)
Odds ratio vs. placebo	0.92	1.59	1.66	2.50	
(95% CI)	(0.26, 3.29)	(0.45, 5.69)	(0.47, 5.92)	(0.70, 8.91)	
20-day rolling averages, *n* (%) ^d^	7 (28.0%)	6 (25.0%)	10 (40.0%)	12 (46.2%)	7 (28.0%)
Odds ratio vs. placebo	1.03	0.93	1.68	2.90 ^d^	
(95% CI)	(0.26, 4.04)	(0.23, 3.79)	(0.45, 6.24)	(0.77, 10.93)	

CI: confidence interval; NPRS: Numerical Pain Rating Scale; SD: standard deviation; TTX: tetrodotoxin; BID: twice daily; QD: once daily; *n*: number of subjects. Pain measured with NPRS. Odds ratios, 95% confidence intervals, and *p*-values were obtained from a logistic regression model by visit with treatment, gender, and baseline numerical pain scale score as covariates. Rolling day averages are based on consecutive days. ^a^ Statistically significant at *p* = 0.072. ^b^ Statistically significant at *p* = 0.059. ^c^ Statistically significant at *p* = 0.027. ^d^ Statistically significant at *p* = 0.071.

**Table 6:** Adverse events.

	TTX Dosage				
	7.5 µg BID (N = 25)	15 µg BID (N = 24)	30 µg QD (N = 25)	30 µg BID (N = 26)	Placebo (N = 25)
Patients with ≥1 AE	21 (84.0)	22 (91.7)	20 (80.0)	24 (92.3)	18 (72.0)
Common AEs, *n* (%) ^a^					
Paresthesia oral	4 (16.0)	9 (37.5)	10 (40.0)	11 (42.3)	3 (12.0)
Hypoesthesia oral	5 (20.0)	7 (29.2)	6 (24.0)	10 (38.5)	3 (12.0)
Paresthesia	5 (20.0)	7 (29.2)	5 (20.0)	7 (26.9)	6 (24.0)
Headache	6 (24.0)	3 (12.5)	1 (4.0)	9 (34.6)	5 (20.0)
Dizziness	3 (12.0)	4 (16.7)	3 (12.0)	8 (30.8)	5 (20.0)
Fatigue	4 (16.0)	5 (20.8)	5 (20.0)	3 (11.5)	4 (16.0)
Nausea	1 (4.0)	5 (20.8)	1 (4.0)	6 (23.1)	6 (24.0)
Pain in extremity	1 (4.0)	5 (20.8)	4 (16.0)	3 (11.5)	2 (8.0)
SAEs, *n* (%)					
Metastatic colon cancer	0	0	0	0	1 (4.0)
Metastatic bladder cancer	0	1 (4.2)	0	0	0
Prostate cancer	0	1 (4.2)	0	0	0
Viral upper respiratory tract infection	0	0	0	1 (3.8)	0
Patients Reporting an AE by Severity, *n* (%)					
Mild	14 (56.0)	12 (50.0)	13 (52.0)	12 (46.2)	9 (36.0)
Moderate	7 (28.0)	9 (37.5)	7 (28.0)	8 (30.8)	9 (36.0)
Severe	0	1 (4.2)	0	1 (3.8)	1 (4.0)
Life Threatening	0	0	0	0	0
Death	0	1 (4.2) Unrelated	0	0	0
Patients Reporting an AE and Relationship to Treatment, *n* (%)					
Not related	6 (24.0)	5 (20.8)	2 (8.0)	4 (15.4)	4 (16.0)
Unlikely related	0	1 (4.2)	0	2 (7.7)	1 (4.0)
Possibly related	8 (32.0)	9 (37.5)	5 (20.0)	9 (34.6)	10 (40.0)
Related	7 (28.0)	7 (29.2)	13 (52.0)	9 (34.6)	3 (12.0)

AE: adverse event; SAE: serious adverse event; TTX: Tetrodotoxin; BID: twice daily; QD: once daily; *n*: number of subjects. All AEs were coded using the Medical Dictionary for Regulatory Activities, version 14.0. ^a^ AEs with >10% occurrence in safety population.

**Table 7:** Onset and duration of hypoesthesia and paresthesia related AEs.

	TTX Dosage				
AE	7.5 µg BID (N = 25)	15 µg BID (N = 24)	30 µg QD (N = 25)	30 µg BID (N = 26)	Placebo (N = 25)
Hypoesthesia, *n*	3	1	7	1	0
Median Onset (h:mm)	0:10	1:38	0:07	0:16	
(Range)	(0:05–0:25)	(1:38–1:38)	(0:02–3:12)	(0:16–0:16)	
Median Duration (h:mm)	0:40	0:12	3:00	1:10	
(Range)	(0:35–0:40)	(0:12–0:12)	(2:30–22:10)	(1:10–1:10)	
Hypoesthesia Oral, *n*	46	38	25	25	4
Median Onset (h:mm)	0:21	0:27	0:19	0:15	0:07
(Range)	(0:00–3:35)	(0:07–7:03)	(0:00–1:30)	(0:05–6:25)	(0:02–1:27)
Median Duration (h:mm)	0:39	1:15	2:51	1:30	4:22
(Range)	(0:05–53:50)	(0:05–5:15)	(0:10–12:44)	(0:15–73:30)	(0:10–55:45)
Paresthesia, *n*	21	14	12	12	9
Median Onset (h:mm)	0:30	0:34	0:21	0:16	0:26
(Range)	(0:00–6:37)	(0:00–3:05)	(0:04–1:13)	(0:00–2:56)	(0:08–3:26)
Median Duration (h:mm)	1:43	0:27	0:50	1:45	0:36
(Range)	(0:03–48:00)	(0:01–59:35)	(0:10–5:28)	(0:05–12:00)	(0:15–4:00)
Paresthesia Oral, *n*	21	28	23	55	8
Median Onset (h:mm)	0:19	0:22	0:24	0:44	0:10
(Range)	(0:05–0:51)	(0:00–14:34)	(0:00–3:18)	(0:00–17:50)	(0:08–0:23)
Median Duration (h:mm)	0:22	0:39	0:40	1:09	0:32
(Range)	(0:04–3:30)	(0:02–3:20)	(0:01–11:00)	(0:01–4:52)	(0:10–2:40)

AE: adverse event; TTX: Tetrodotoxin; BID: twice daily; QD: once daily; *n*: number of events; h: hours; mm: minutes. Hypoesthesia oral includes AEs of hypoesthesia oral, hypoesthesia facial, pharyngeal hypoesthesia and hypoesthesia teeth.

## Data Availability

Individual de-identified patient data and related documents (e.g., study protocol, statistical analysis plan) will be shared by request from any qualified investigator for the sole purpose of replicating procedures and results presented in the article and as long as data transfer is in agreement with local legislation on general data protection. Additional information regarding the clinical trial can be found at ClinicalTrials.gov identifier NCT01655823.

## Supplementary Materials

The following are available online at https://www.mdpi.com/2072-6651/13/4/235/s1, Figure S1: SF-36 Secondary Outcome Measures, Figure S2: EORTC CIPN20 Secondary Outcome Measures, Figure S3: Numerical Pain Rating Scale (NPRS) Scores, Table S1: Patient Global Impression of Change (PGIC) at Day 28, Table S2: Study Adverse Events.
