# Supplementary material for: Tetrodotoxin for Chemotherapy-Induced Neuropathic Pain: A Randomized, Double-Blind, Placebo-Controlled, Parallel-Dose Finding Trial
Source: Toxins (Basel). 2021 Mar 25;13(4):235. doi: 10.3390/toxins13040235 (PMC8064362; doi:10.3390/toxins13040235)
Supplement: Supplementary file 1 [file toxins-13-00235-s001.pdf]

Supplementary Materials: Tetrodotoxin for Chemotherapy-Induced Neuropathic Pain: A Randomized, Double-Blind, Placebo-Controlled, Parallel-Dose Finding Trial

Samuel A. Goldlust, Mojgan Kavoosi, Jennifer Nezzer, Mehran Kavoosi, Walter Korz and Kenneth Deck

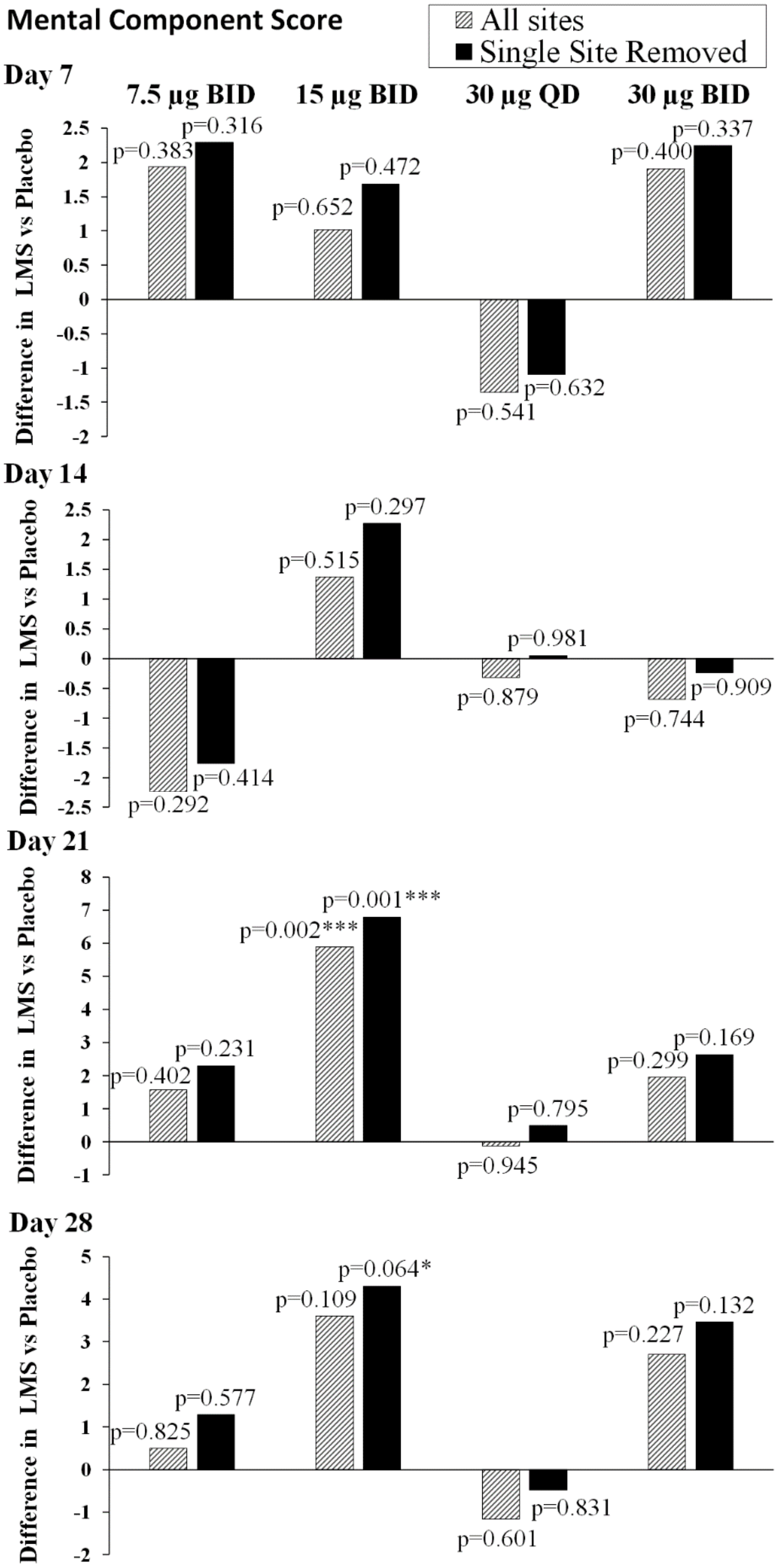
Mental Component Score
All sites
Single Site Removed
Day 7
7.5 µg BID
15 µg BID
30 µg QD
30 µg BID
Difference in LMS vs Placebo
p=0.383
p=0.316
p=0.652
p=0.472
p=0.541
p=0.632
p=0.400
p=0.337
Day 14
p=0.292
p=0.414
p=0.515
p=0.297
p=0.879
p=0.981
p=0.744
p=0.909
Day 21
p=0.402
p=0.231
p=0.002***
p=0.001***
p=0.945
p=0.795
p=0.299
p=0.169
Day 28
p=0.825
p=0.577
p=0.109
p=0.064*
p=0.601
p=0.831
p=0.227
p=0.132

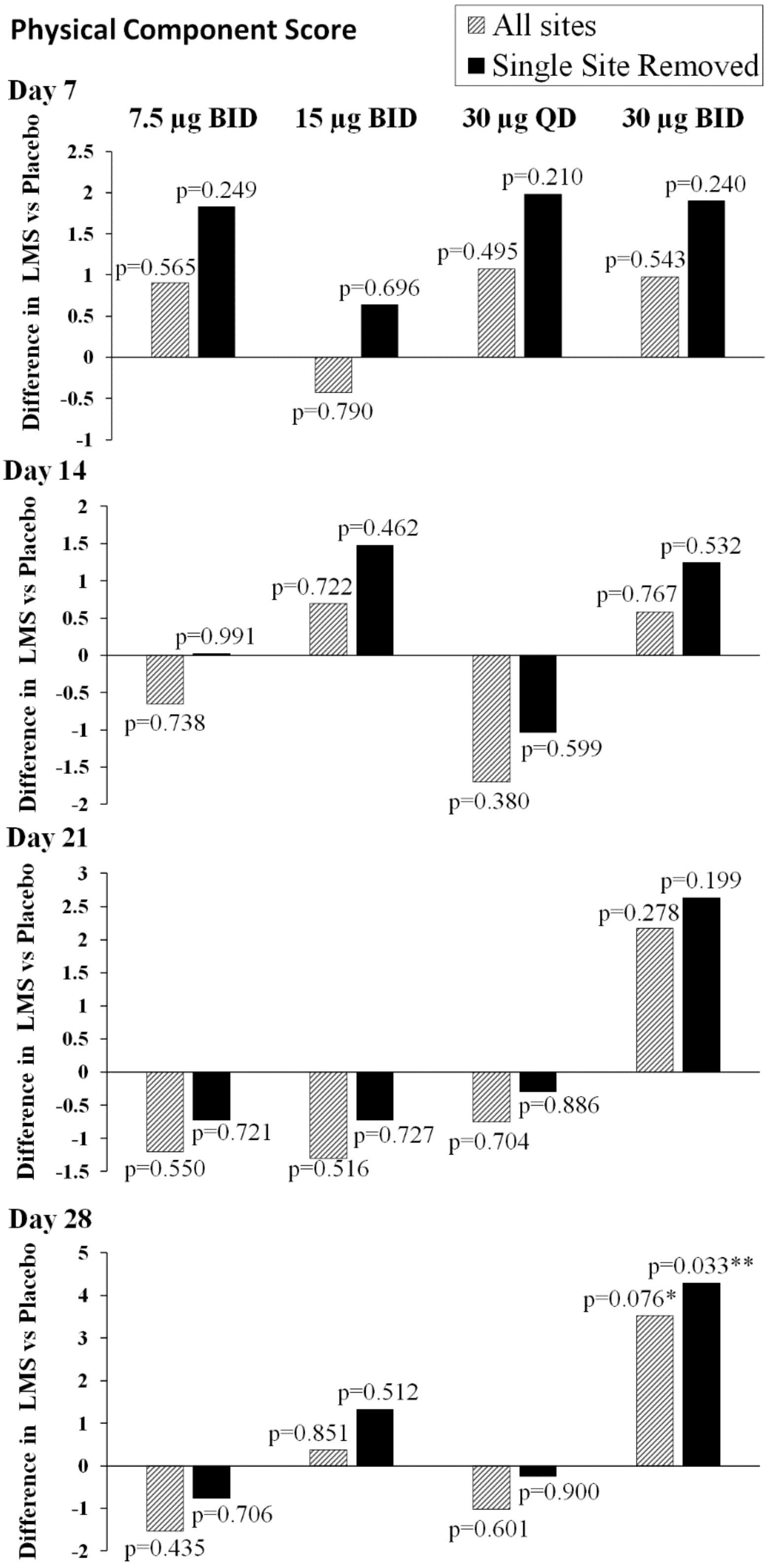
Physical Component Score
All sites
Single Site Removed
Day 7
7.5 µg BID
15 µg BID
30 µg QD
30 µg BID
Difference in LMS vs Placebo
p=0.565
p=0.249
p=0.790
p=0.696
p=0.495
p=0.210
p=0.543
p=0.240
Day 14
p=0.738
p=0.991
p=0.722
p=0.462
p=0.380
p=0.599
p=0.767
p=0.532
Day 21
p=0.550
p=0.721
p=0.516
p=0.727
p=0.704
p=0.886
p=0.278
p=0.199
Day 28
p=0.435
p=0.706
p=0.851
p=0.512
p=0.601
p=0.900
p=0.076*
p=0.033**

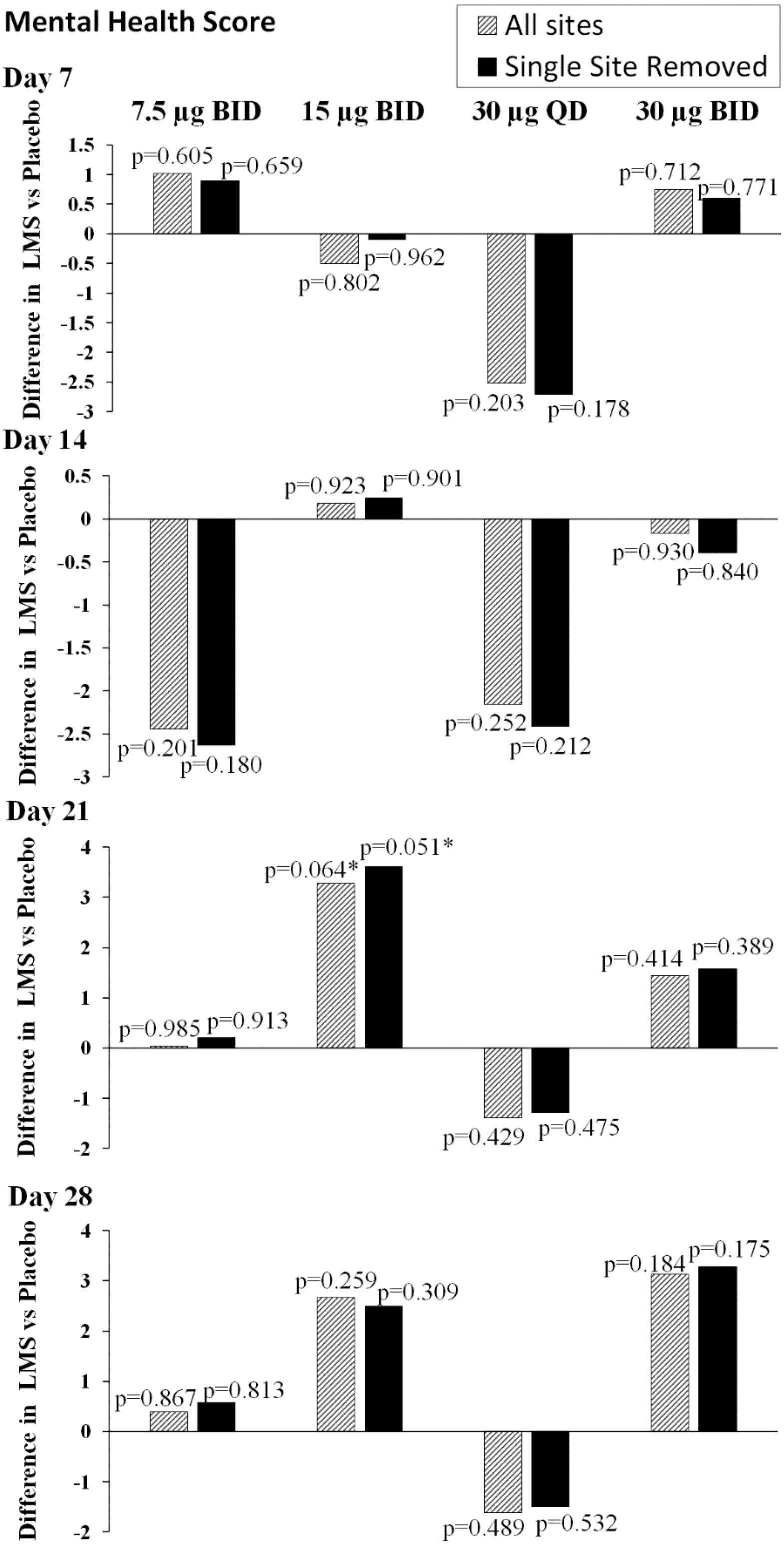
Mental Health Score
All sites
Single Site Removed
Day 7
7.5 µg BID
15 µg BID
30 µg QD
30 µg BID
Difference in LMS vs Placebo
1.5
1
0.5
0
-0.5
-1
-1.5
-2
-2.5
-3
p=0.605
p=0.659
p=0.802
p=0.962
p=0.203
p=0.178
p=0.712
p=0.771
Day 14
Difference in LMS vs Placebo
0.5
0
-0.5
-1
-1.5
-2
-2.5
-3
p=0.201
p=0.180
p=0.923
p=0.901
p=0.252
p=0.212
p=0.930
p=0.840
Day 21
Difference in LMS vs Placebo
4
3
2
1
0
-1
-2
p=0.985
p=0.913
p=0.064*
p=0.051*
p=0.429
p=0.475
p=0.414
p=0.389
Day 28
Difference in LMS vs Placebo
4
3
2
1
0
-1
-2
p=0.867
p=0.813
p=0.259
p=0.309
p=0.489
p=0.532
p=0.184
p=0.175

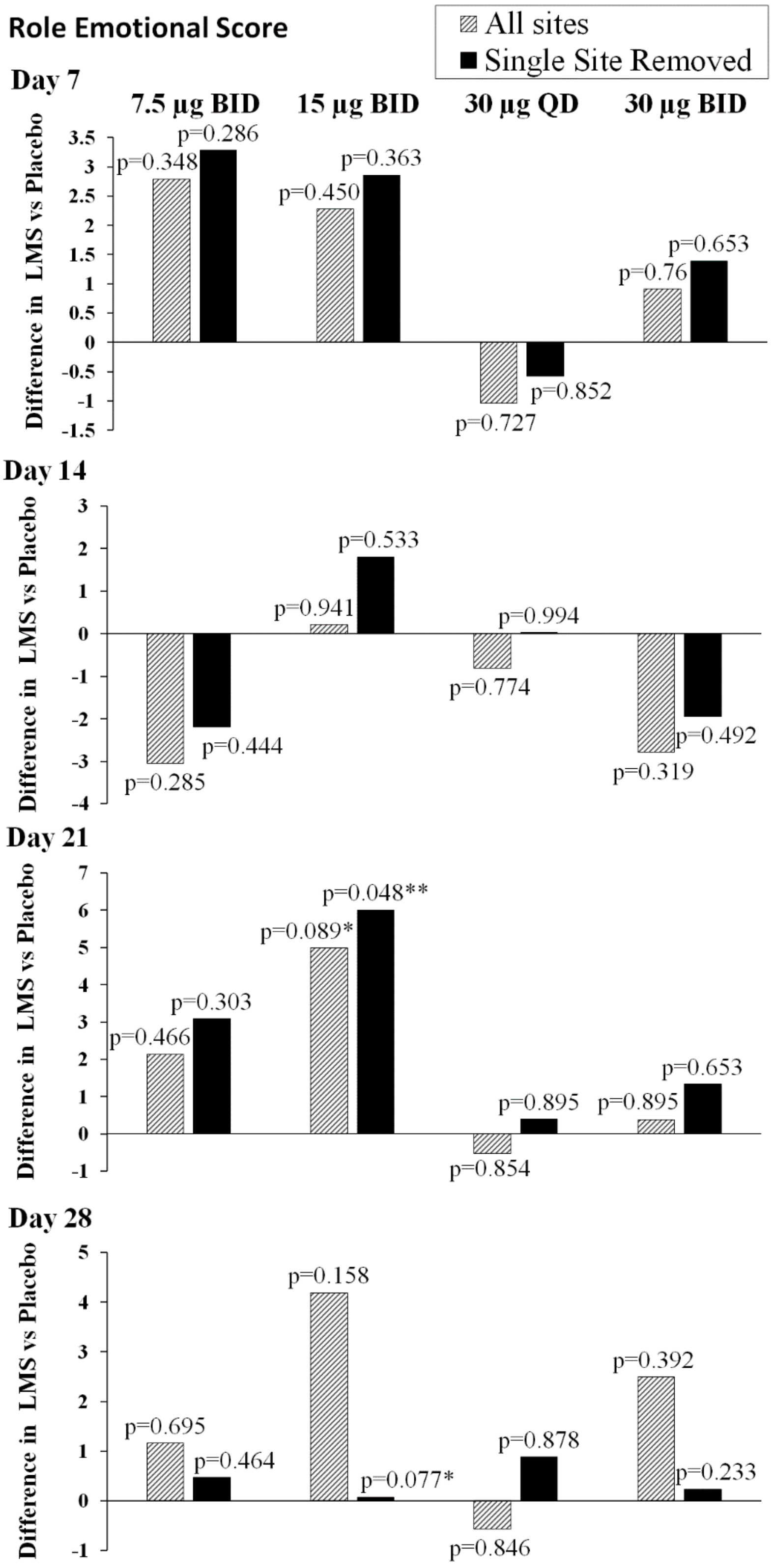
Role Emotional Score
All sites
Single Site Removed
Day 7
7.5 µg BID
15 µg BID
30 µg QD
30 µg BID
Difference in LMS vs Placebo
p=0.348
p=0.286
p=0.450
p=0.363
p=0.727
p=0.852
p=0.76
p=0.653
Day 14
p=0.285
p=0.444
p=0.941
p=0.533
p=0.774
p=0.994
p=0.319
p=0.492
Day 21
p=0.466
p=0.303
p=0.089*
p=0.048**
p=0.854
p=0.895
p=0.895
p=0.653
Day 28
p=0.695
p=0.464
p=0.158
p=0.077*
p=0.846
p=0.878
p=0.392
p=0.233

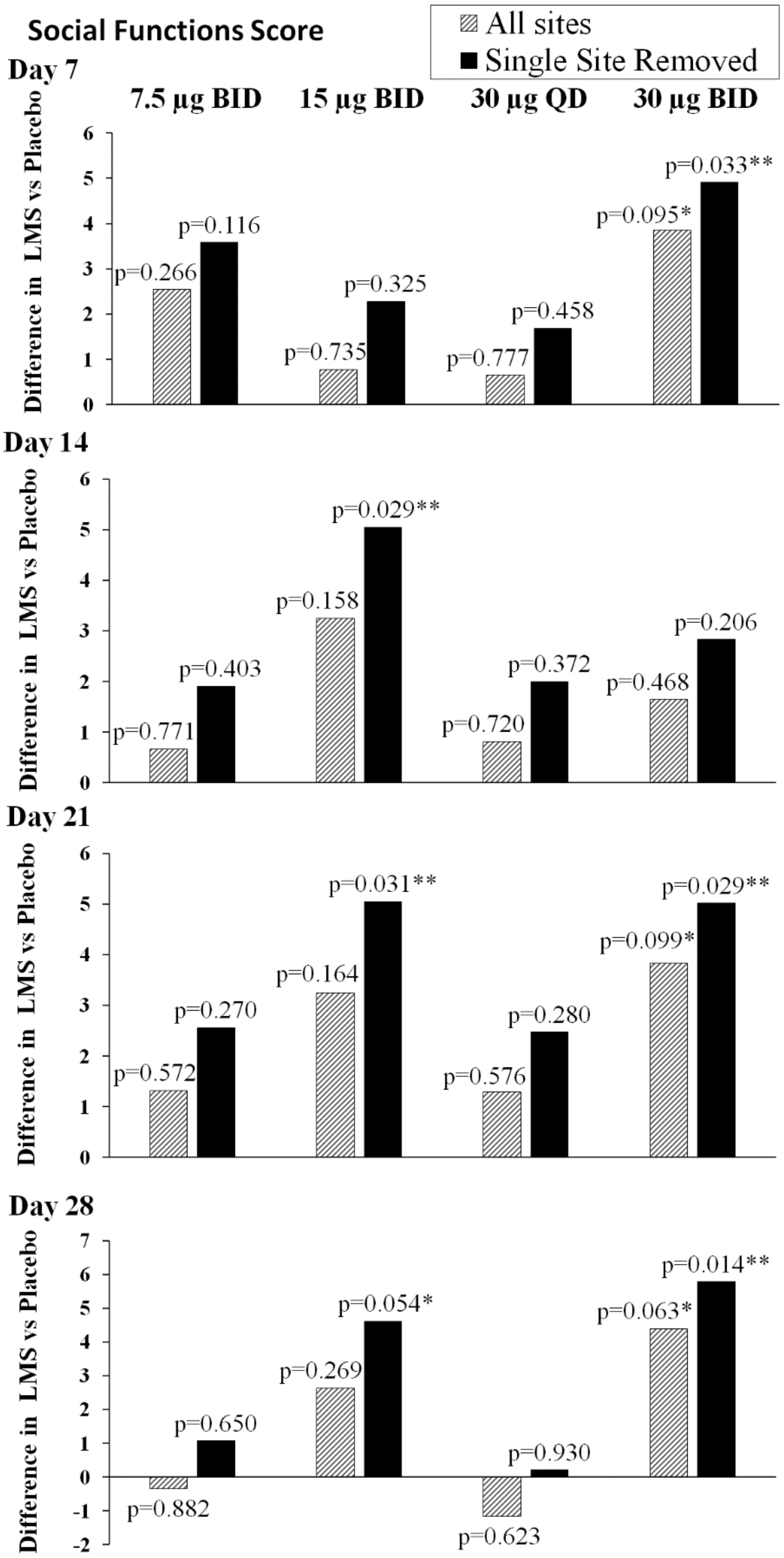
Social Functions Score
All sites
Single Site Removed
Day 7
7.5 µg BID
15 µg BID
30 µg QD
30 µg BID
Difference in LMS vs Placebo
p=0.266
p=0.116
p=0.735
p=0.325
p=0.777
p=0.458
p=0.095*
p=0.033**
Day 14
p=0.771
p=0.403
p=0.158
p=0.029**
p=0.720
p=0.372
p=0.468
p=0.206
Day 21
p=0.572
p=0.270
p=0.164
p=0.031**
p=0.576
p=0.280
p=0.099*
p=0.029**
Day 28
p=0.882
p=0.650
p=0.269
p=0.054*
p=0.623
p=0.930
p=0.063*
p=0.014**

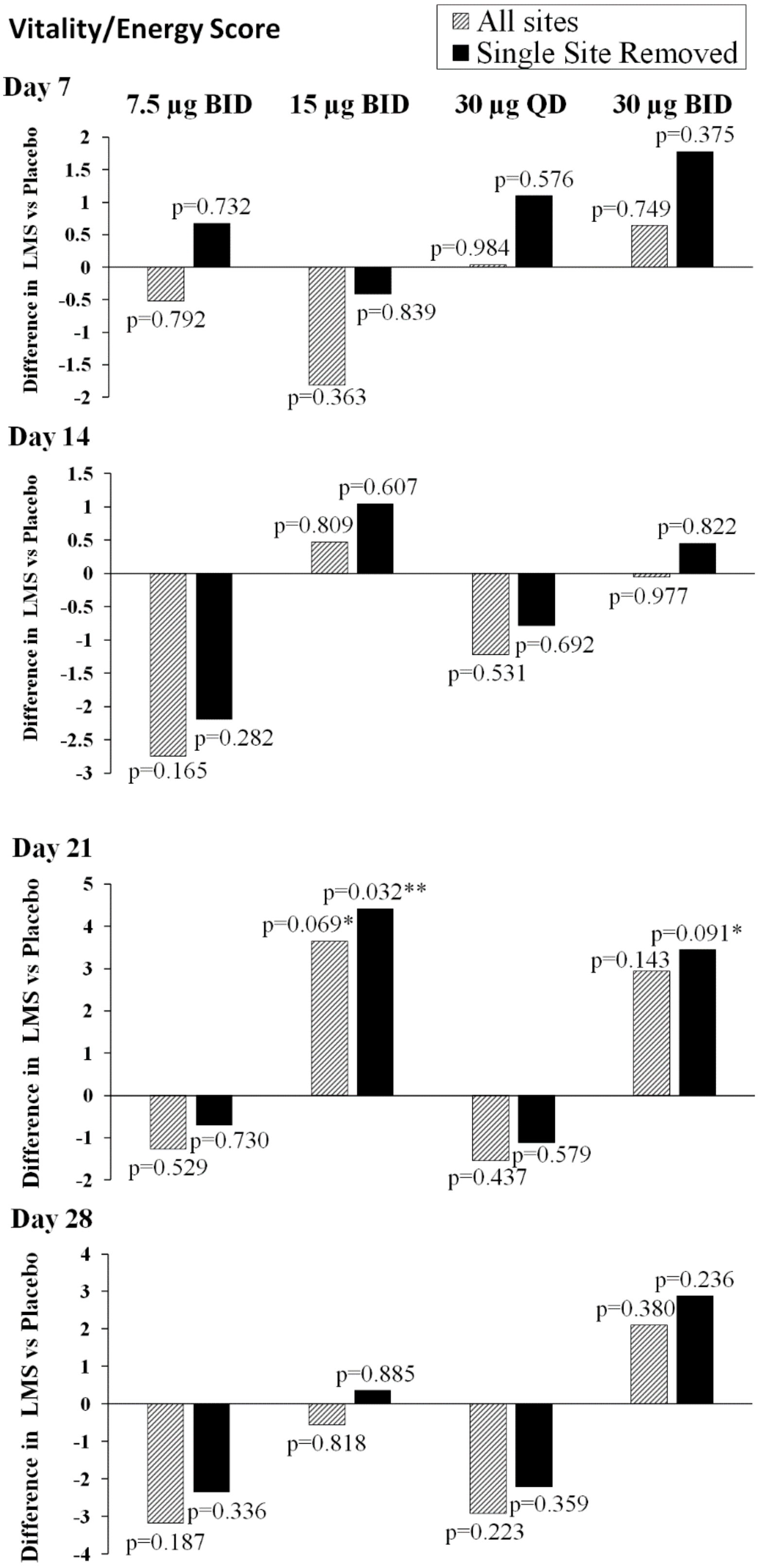
Vitality/Energy Score
All sites
Single Site Removed
Day 7
7.5 µg BID
15 µg BID
30 µg QD
30 µg BID
Difference in LMS vs Placebo
p=0.732
p=0.792
p=0.839
p=0.363
p=0.576
p=0.984
p=0.375
p=0.749
Day 14
p=0.165
p=0.282
p=0.809
p=0.607
p=0.531
p=0.692
p=0.822
p=0.977
Day 21
p=0.529
p=0.730
p=0.069*
p=0.032**
p=0.437
p=0.579
p=0.143
p=0.091*
Day 28
p=0.187
p=0.336
p=0.818
p=0.885
p=0.223
p=0.359
p=0.380
p=0.236

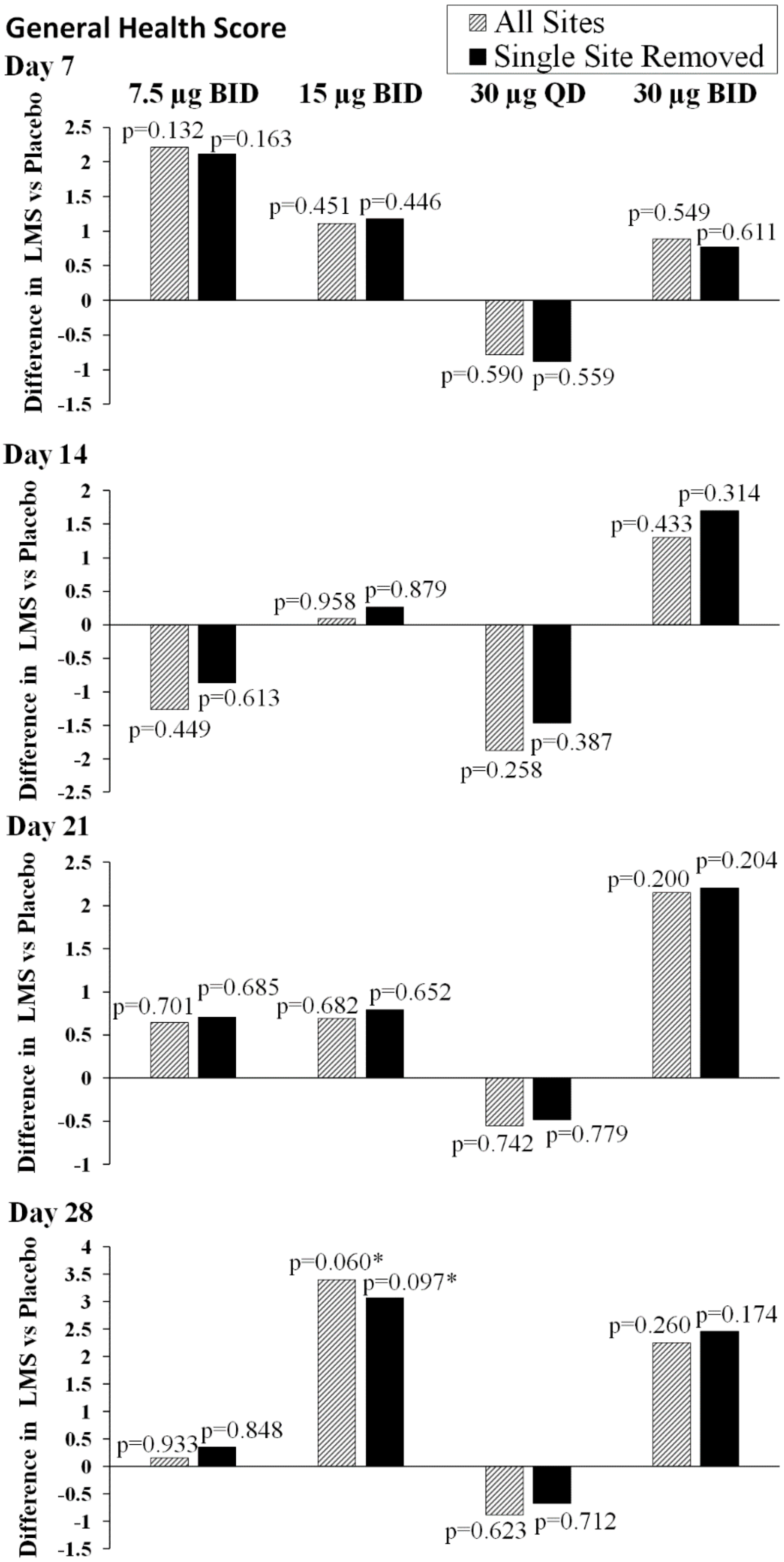
General Health Score
All Sites
Single Site Removed
Day 7
7.5 µg BID
15 µg BID
30 µg QD
30 µg BID
Difference in LMS vs Placebo
p=0.132
p=0.163
p=0.451
p=0.446
p=0.549
p=0.611
p=0.590
p=0.559
Day 14
p=0.314
p=0.433
p=0.958
p=0.879
p=0.613
p=0.449
p=0.387
p=0.258
Day 21
p=0.200
p=0.204
p=0.701
p=0.685
p=0.682
p=0.652
p=0.742
p=0.779
Day 28
p=0.060*
p=0.097*
p=0.260
p=0.174
p=0.933
p=0.848
p=0.623
p=0.712

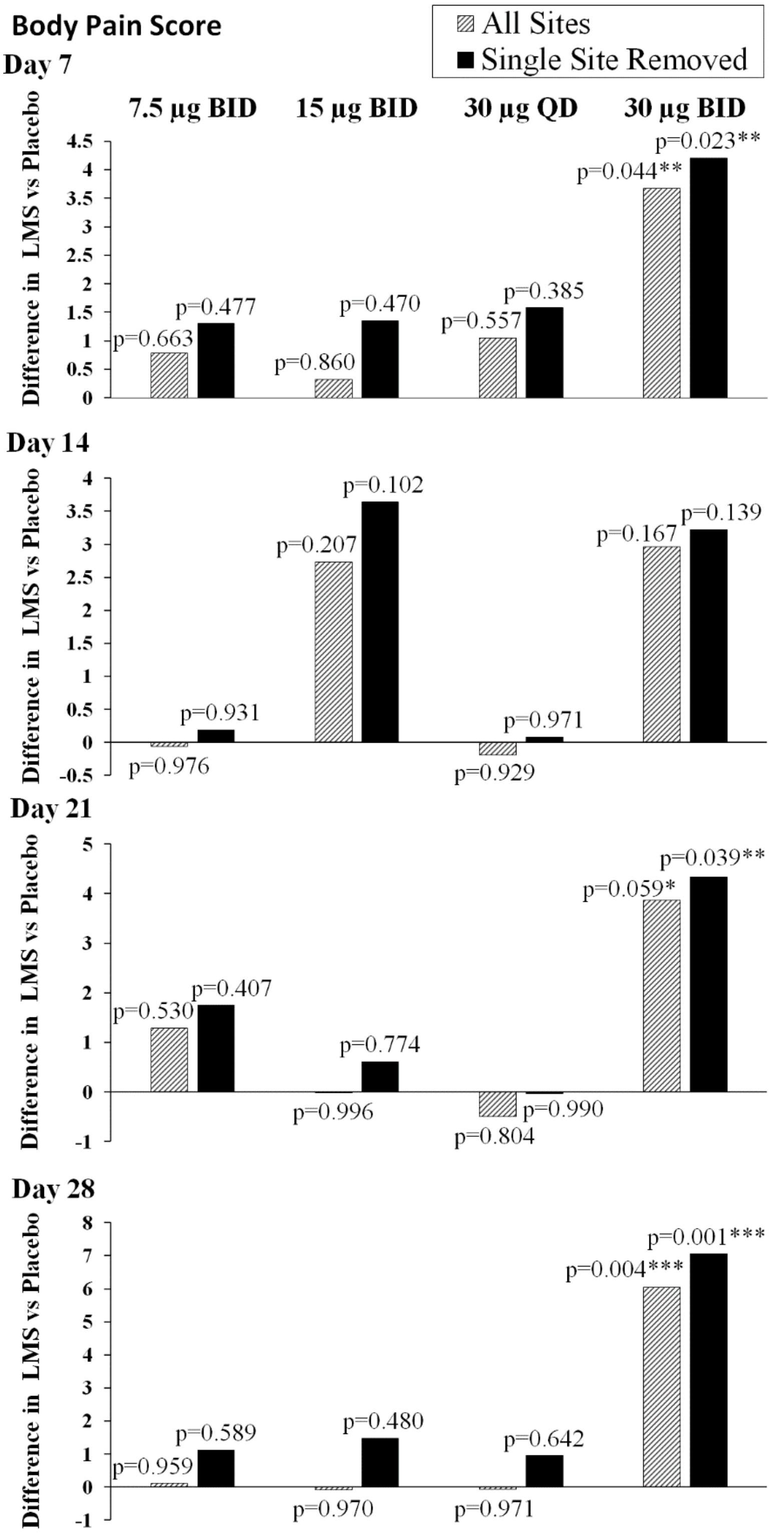
Body Pain Score
All Sites
Single Site Removed
Day 7
7.5 µg BID
15 µg BID
30 µg QD
30 µg BID
Difference in LMS vs Placebo
p=0.663
p=0.477
p=0.860
p=0.470
p=0.557
p=0.385
p=0.044**
p=0.023**
Day 14
p=0.976
p=0.931
p=0.207
p=0.102
p=0.929
p=0.971
p=0.167
p=0.139
Day 21
p=0.530
p=0.407
p=0.996
p=0.774
p=0.804
p=0.990
p=0.059*
p=0.039**
Day 28
p=0.959
p=0.589
p=0.970
p=0.480
p=0.971
p=0.642
p=0.004***
p=0.001***

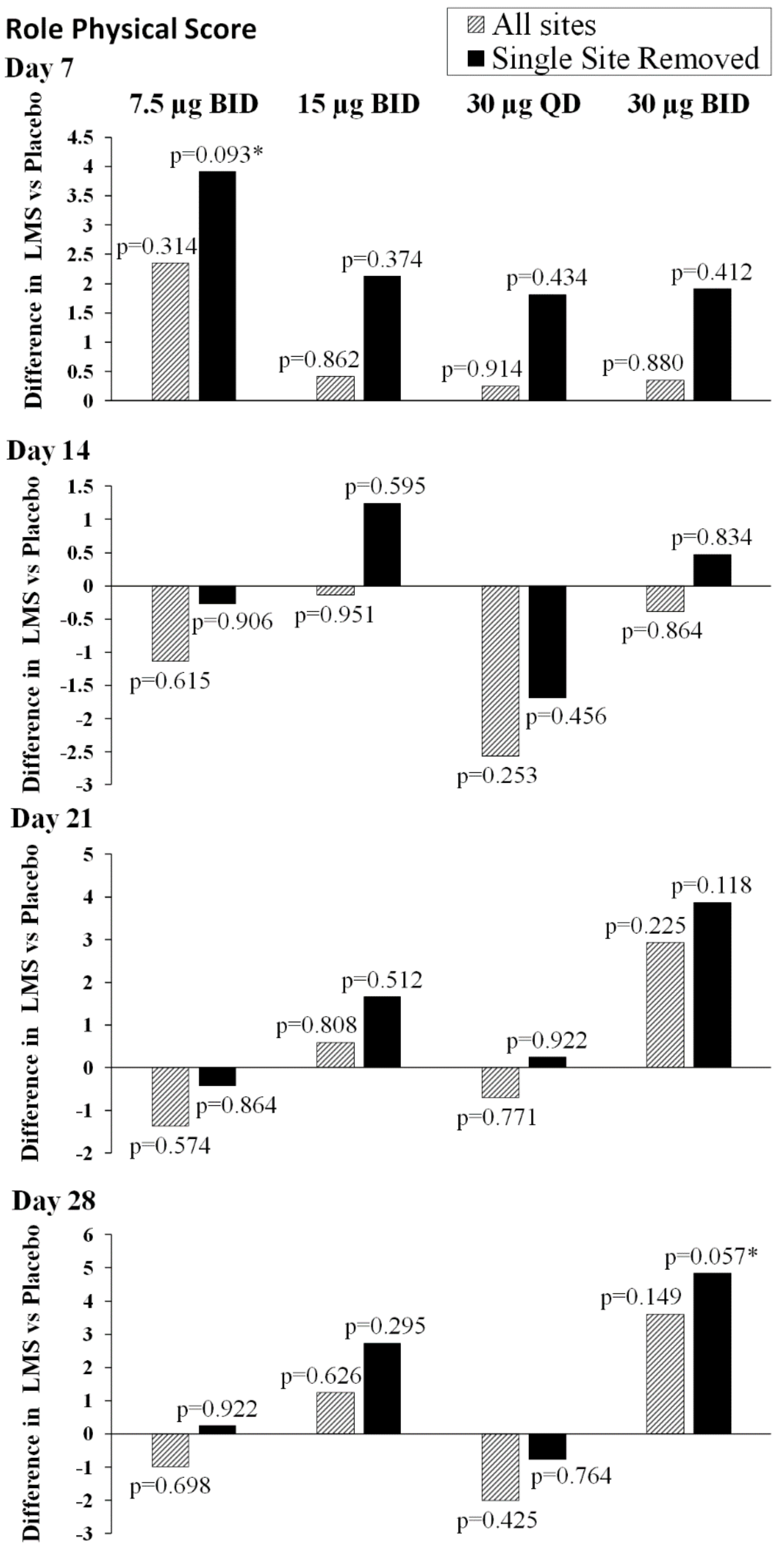
Role Physical Score
All sites
Single Site Removed
Day 7
7.5 µg BID
15 µg BID
30 µg QD
30 µg BID
Difference in LMS vs Placebo
p=0.093*
p=0.314
p=0.374
p=0.434
p=0.412
p=0.862
p=0.914
p=0.880
Day 14
p=0.595
p=0.834
p=0.906
p=0.951
p=0.864
p=0.615
p=0.456
p=0.253
Day 21
p=0.118
p=0.225
p=0.512
p=0.808
p=0.922
p=0.864
p=0.771
p=0.574
Day 28
p=0.057*
p=0.149
p=0.295
p=0.626
p=0.922
p=0.698
p=0.764
p=0.425

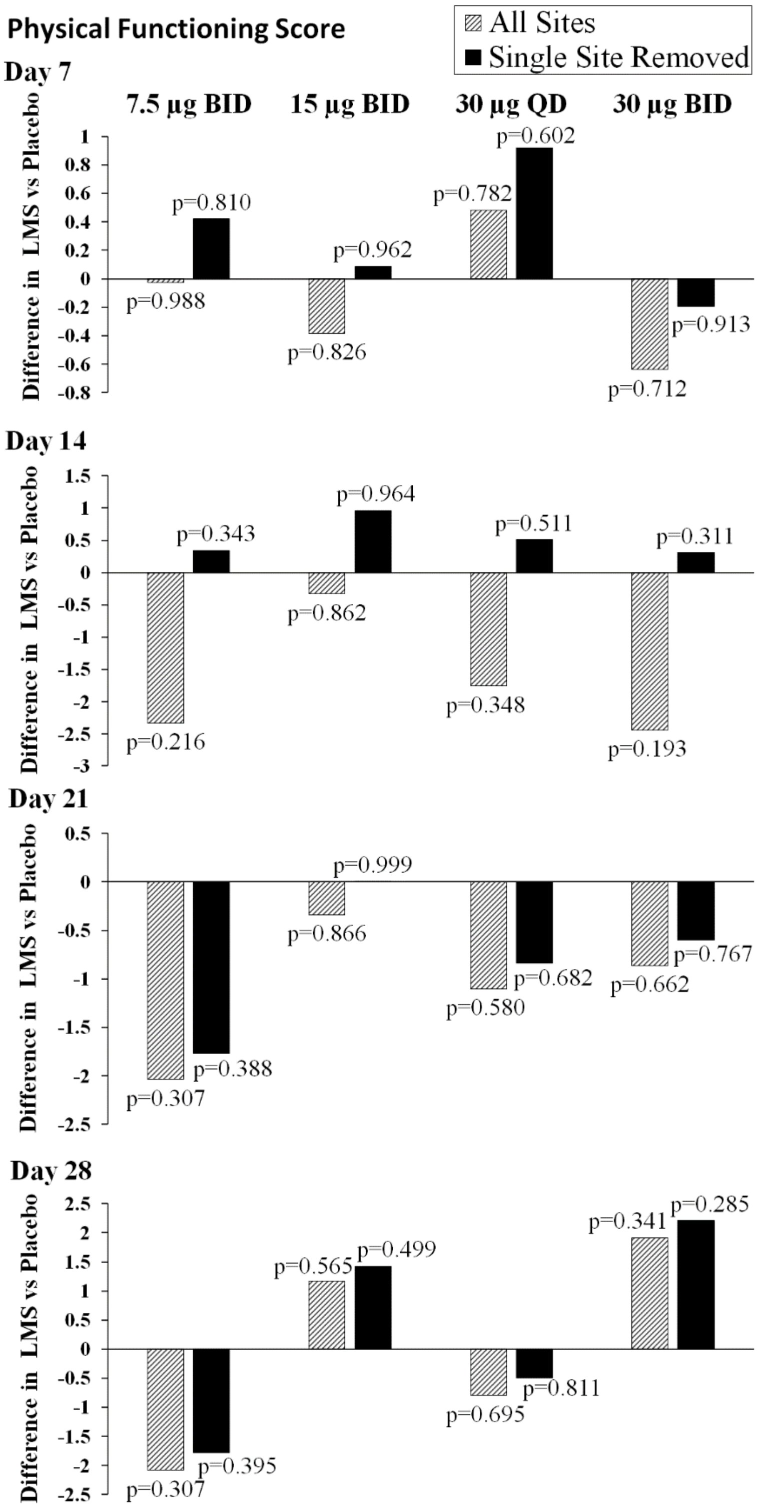

Figure S1. SF-36 Secondary Outcome Measures. The least squares mean (LMS) differences (treatment—placebo) from baseline to Day 7, Day 14, Day 21, and Day 28 are shown. *P*-values for the difference in means are shown with * indicates $p < 0.1$, ** indicates $p < 0.05$, and *** indicates $p < 0.01$.

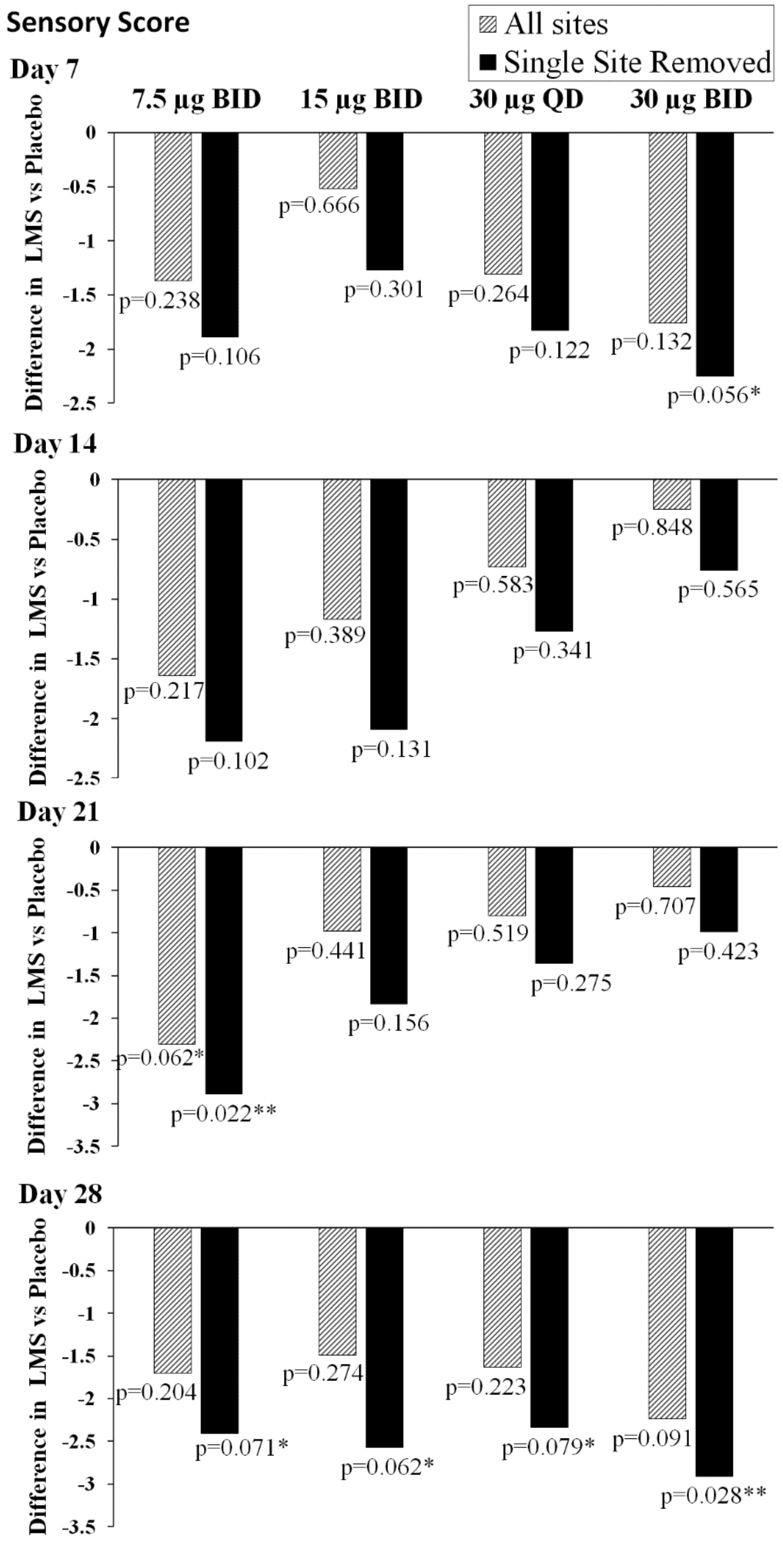
Sensory Score
All sites
Single Site Removed
Day 7
7.5 µg BID
15 µg BID
30 µg QD
30 µg BID
Difference in LMS vs Placebo
0
-0.5
-1
-1.5
-2
-2.5
p=0.238
p=0.106
p=0.666
p=0.301
p=0.264
p=0.122
p=0.132
p=0.056*
Day 14
p=0.217
p=0.102
p=0.389
p=0.131
p=0.583
p=0.341
p=0.848
p=0.565
Day 21
-3
-3.5
p=0.062*
p=0.022**
p=0.441
p=0.156
p=0.519
p=0.275
p=0.707
p=0.423
Day 28
p=0.204
p=0.071*
p=0.274
p=0.062*
p=0.223
p=0.079*
p=0.091
p=0.028**

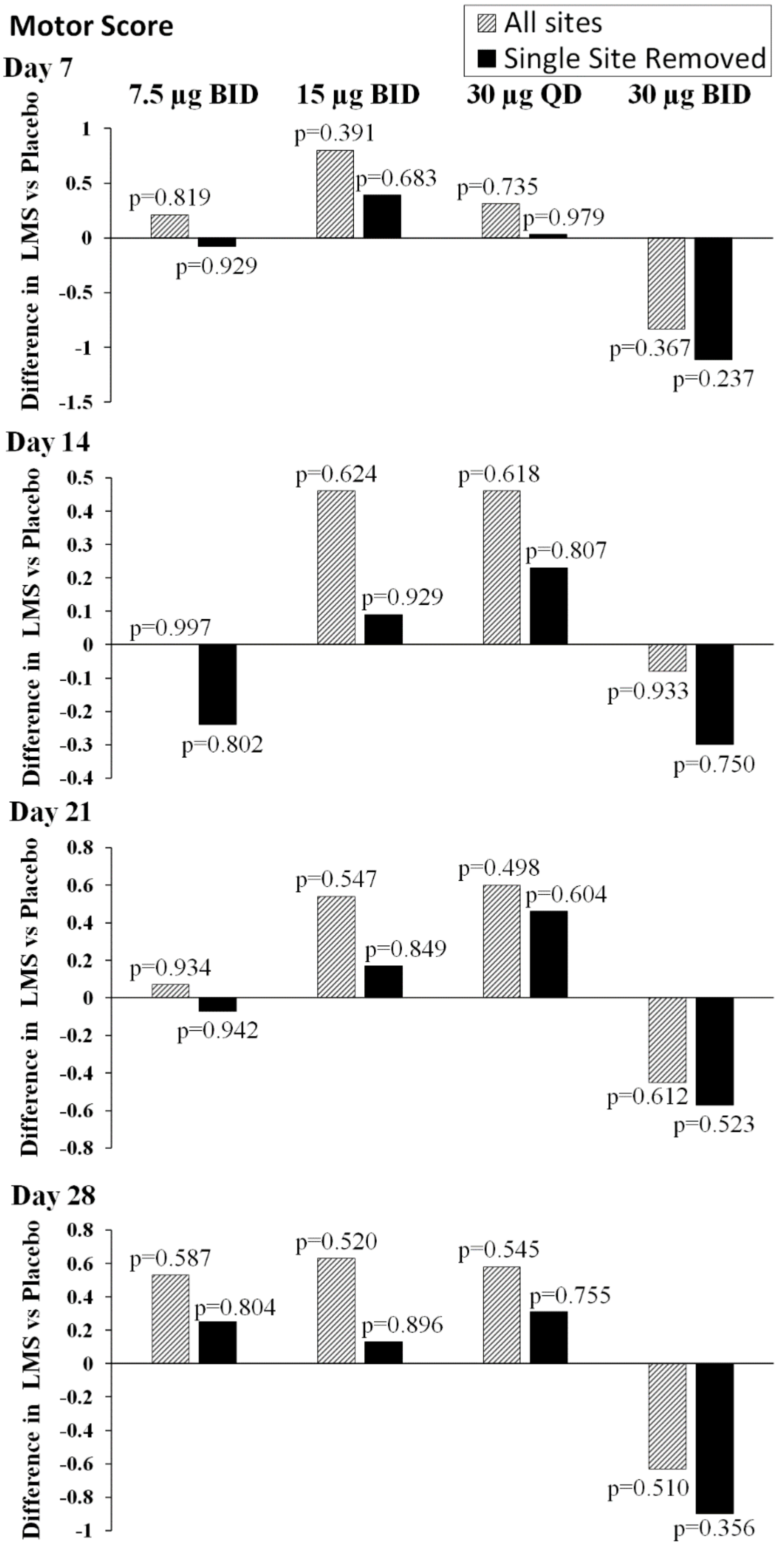
Motor Score
All sites
Single Site Removed
Day 7
7.5 µg BID
15 µg BID
30 µg QD
30 µg BID
Difference in LMS vs Placebo
p=0.819
p=0.929
p=0.391
p=0.683
p=0.735
p=0.979
p=0.367
p=0.237
Day 14
p=0.997
p=0.802
p=0.624
p=0.929
p=0.618
p=0.807
p=0.933
p=0.750
Day 21
p=0.934
p=0.942
p=0.547
p=0.849
p=0.498
p=0.604
p=0.612
p=0.523
Day 28
p=0.587
p=0.804
p=0.520
p=0.896
p=0.545
p=0.755
p=0.510
p=0.356

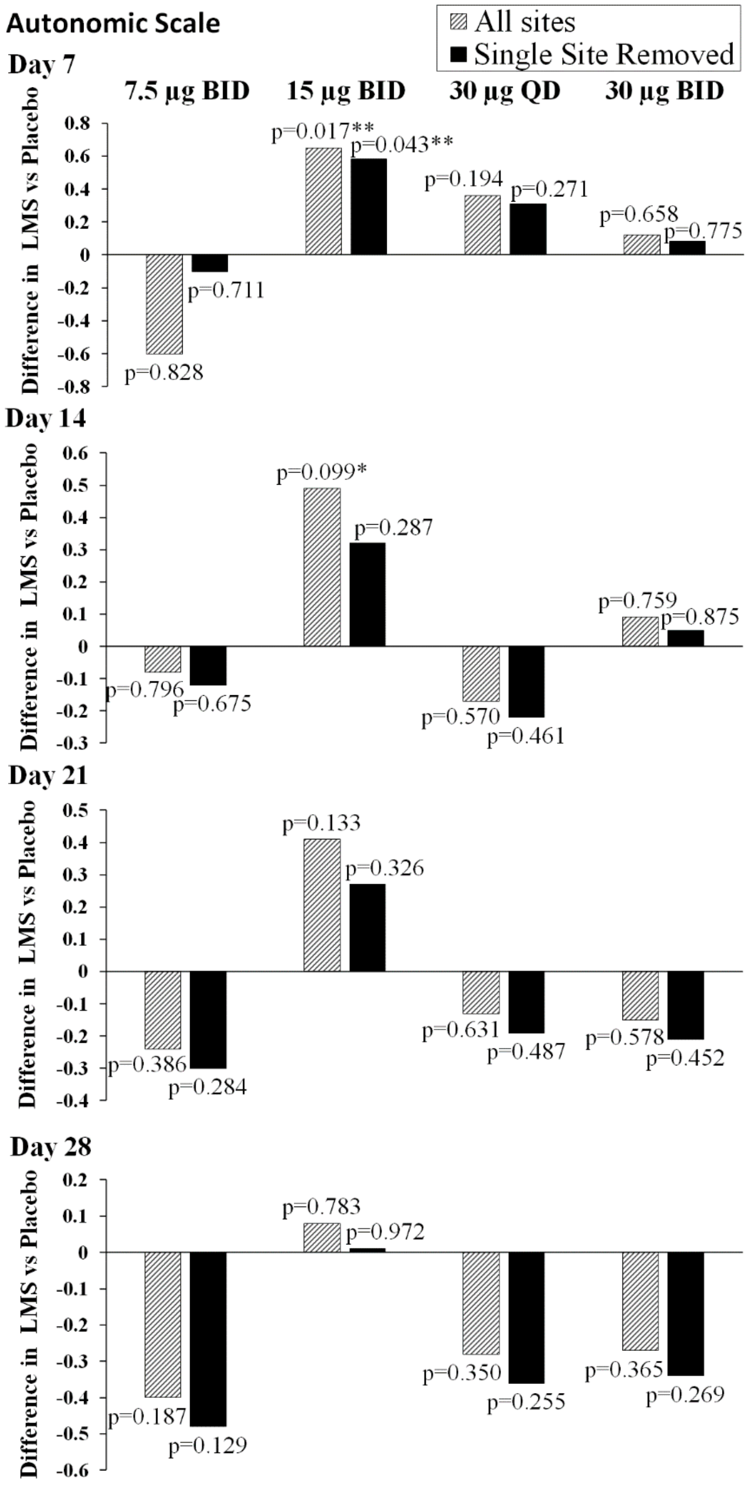

Figure S2: EORTC CIPN20 Secondary Outcome Measures. The least squares mean (LMS) differences (treatment – placebo) from baseline to Day 7, Day 14, Day 21, and Day 28 are shown. *P*-values for the difference in means are shown with * indicates $p < 0.1$, ** indicates $p < 0.05$, and *** indicates $p < 0.01$.

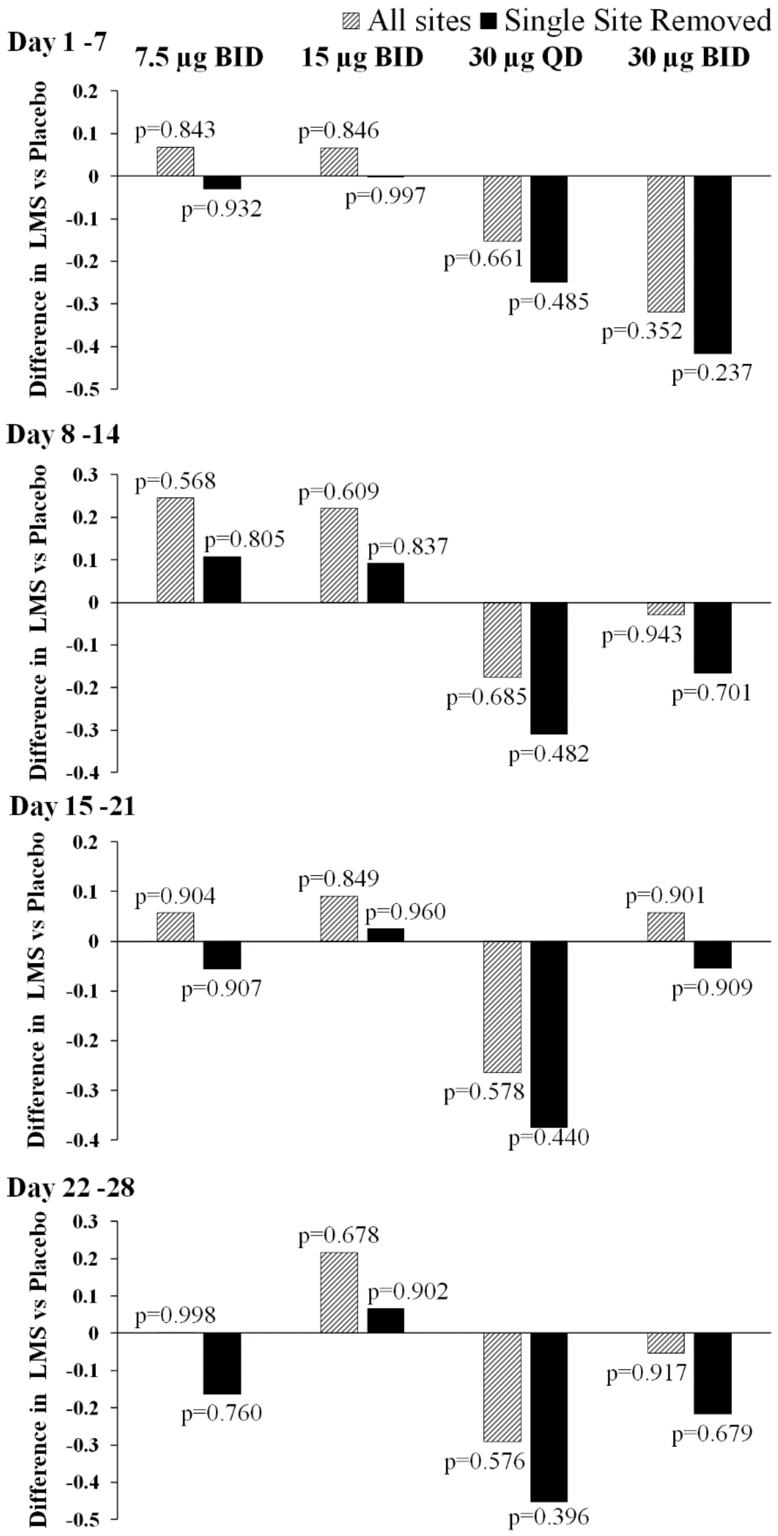

Figure S3: Numerical Pain Rating Scale (NPRS) Scores. The change from baseline in average least squares mean (LMS) differences (treatment – placebo) for each week are shown. P-values represent the significant difference from placebo.

Table S1: Patient Global Impression of Change (PGIC) at Day 28

	TTX Dosage				
	**7.5 μg BID (N = 25)**	**15 μg BID (N = 24)**	**30 μg QD (N = 25)**	**30 μg BID (N = 26)**	**Placebo (N = 25)**
**Since starting this study:**	***n* (%)**	***n* (%)**	***n* (%)**	***n* (%)**	***n* (%)**
**Overall QoL:**					
Very much worse	0	0	0	0	1 (4.0%)
Moderately worse	0	0	1 (4.0%)	1 (3.8%)	1 (4.0%)
A little worse	1 (4.0%)	2 (8.3%)	2 (8.0%)	1 (3.8%)	0
About the same	11 (44.0%)	8 (33.3%)	9 (36.0%)	9 (34.6%)	9 (36.0%)
A little better	5 (20.0%)	5 (20.8%)	2 (8.0%)	2 (7.7%)	5 (20.0%)
Moderately better	5 (20.0%)	6 (25.0%)	6 (24.0%)	10 (38.5%)	5 (20.0%)
Very much better	1 (4.0%)	3 (12.5%)	3 (12.0%)	2 (7.7%)	4 (16.0%)
p-value [a]	0.148	0.402	0.927	1.000	
**Physical Condition:**					
Very much worse	0	0	0	0	0
Moderately worse	0	1 (4.2%)	1 (4.0%)	0	2 (8.0%)
A little worse	1 (4.0%)	1 (4.2%)	1 (4.0%)	2 (7.7%)	0
About the same	13 (52.0%)	8 (33.3%)	10 (40.0%)	8 (30.8%)	11 (44.0%)
A little better	3 (12.0%)	8 (33.3%)	3 (12.0%)	5 (19.2%)	5 (20.0%)
Moderately better	5 (20.0%)	4 (16.7%)	5 (20.0%)	9 (34.6%)	3 (12.0%)
Very much better	1 (4.0%)	2 (8.3%)	3 (12.0%)	1 (3.8%)	4 (16.0%)
p-value [a]	0.433	0.219	0.943	0.385	
**Emotional State:**					
Very much worse	0	1 (4.2%)	1 (4.0%)	0	0
Moderately worse	0	0	0	1 (3.8%)	0
A little worse	0	0	1 (4.0%)	0	4 (16.0%)
About the same	17 (68.0%)	15 (62.5%)	14 (56.0%)	12 (46.2%)	11 (44.0%)
A little better	3 (12.0%)	5 (20.8%)	2 (8.0%)	4 (15.4%)	4 (16.0%)
Moderately better	2 (8.0%)	2 (8.3%)	4 (16.0%)	3 (11.5%)	2 (8.0%)
Very much better	1 (4.0%)	1 (4.2%)	1 (4.0%)	5 (19.2%)	4 (16.0%)
p-value [a]	0.982	0.898	0.451	0.370	
**Enjoy Social Life:**					
Very much worse	0	0	0	0	0
Moderately worse	0	0	0	0	1 (4.0%)
A little worse	0	0	0	1 (3.8%)	1 (4.0%)
About the same	16 (64.0%)	15 (62.5%)	15 (60.0%)	12 (46.2%)	12 (48.0%)
A little better	2 (8.0%)	5 (20.8%)	3 (12.0%)	3 (11.5%)	4 (16.0%)
Moderately better	4 (16.0%)	3 (12.5%)	3 (12.0%)	5 (19.2%)	3 (12.0%)
Very much better	1 (4.0%)	1 (4.2%)	2 (8.0%)	4 (15.4%)	4 (16.0%)
p-value [a]	0.612	0.218	0.636	0.849	
**Numbness, Tingling or Pain in Hands or Feet:**					
Very much worse	0	0	1 (4.0%)	1 (3.8%)	1 (4.0%)
Moderately worse	0	1 (4.2%)	2 (8.0%)	1 (3.8%)	2 (8.0%)
A little worse	1 (4.0%)	2 (8.3%)	1 (4.0%)	3 (11.5%)	0
About the same	10 (40.0%)	8 (33.3%)	8 (32.0%)	6 (23.1%)	9 (36.0%)

A little better	4 (16.0%)	7 (29.2%)	3 (12.0%)	5 (19.2%)	7 (28.0%)
Moderately better	6 (24.0%)	6 (25.0%)	3 (12.0%)	6 (23.1%)	2 (8.0%)
Very much better	2 (8.0%)	0	5 (20.0%)	3 (11.5%)	4 (16.0%)
p-value [a]	0.691	0.165	0.342	1.000	
**Believe Receiving Active Agent:**					
Yes	15 (60.0%)	15 (62.5%)	14 (56.0%)	22 (84.6%)	13 (52.0%)
No	8 (32.0%)	9 (37.5%)	8 (32.0%)	3 (11.5%)	12 (48.0%)

QoL: quality of life; BID: twice daily; QD: once daily; *n*: number of subjects. [a] Nominal *p*-values were obtained from Cochran-Mantel-Haenszel tests for comparing difference between active treatment and placebo cohorts.

Table S2: Study Adverse Events

**System Organ Class**<br>**Preferred Term**	**TTX**<br>**7.5 µg BID**<br>**(N = 25)**<br>***n* (%)**		**TTX**<br>**15 µg BID**<br>**(N = 24)**<br>***n* (%)**		**TTX**<br>**30 µg QD**<br>**(N = 25)**<br>***n* (%)**		**TTX**<br>**30 µg BID**<br>**(N = 26)**<br>***n* (%)**		**Placebo**<br>**(N = 25)**<br>***n* (%)**	
**Number of Subjects with ≥1 AE**	21 (84.0%)		22 (91.7%)		20 (80.0%)		24 (92.3%)		18 (72.0%)	
**Nervous system disorders**										
Paraesthesia oral	4 (16.0%)		9 (37.5%)		10 (40.0%)		11 (42.3%)		3 (12.0%)	
	3× Mild<br>1× Moderate	2× Possibly<br>2× Related	8× Mild<br>1× Moderate	6× Possibly<br>3× Related	10× Mild	3× Possibly<br>7× Related	11× Mild	2× Not Related<br>5× Possibly<br>4× Related	3× Mild	2× Possibly<br>1× Related
Hypoaesthesia oral	5 (20.0%)		7 (29.2%)		6 (24.0%)		10 (38.5%)		3 (12.0%)	
	4× Mild<br>1× Moderate	4× Possibly<br>1× Related	6× Mild<br>1× Moderate	5× Possibly<br>2× Related	6× Mild	3× Possibly<br>3× Related	9× Mild<br>1× Moderate	1× Not Related<br>3× Possibly<br>6× Related	3× Mild	2× Possibly<br>1× Related
Paraesthesia	5 (20.0%)		7 (29.2%)		5 (20.0%)		7 (26.9%)		6 (24.0%)	
	4× Mild<br>1× Moderate	1× Possibly<br>4× Related	6× Mild<br>1× Moderate	5× Possibly<br>2× Related	5× Mild	1× Not Related<br>4× Related	6× Mild<br>1× Severe	1× Not Related<br>3× Possibly<br>3× Related	5× Mild<br>1× Moderate	1× Not Related<br>4× Possibly<br>1× Related
Headache	6 (24.0%)		3 (12.5%)		1 (4.0%)		9 (34.6%)		5 (20.0%)	
	5× Mild<br>1× Moderate	2× Unlikely<br>3× Possibly<br>1× Related	3 × Mild	1× Unlikely<br>1× Possibly<br>1× Related	1× Mild	1× Not Related	8× Mild<br>1× Moderate	4× Not Related<br>2× Possibly<br>3× Related	4× Mild<br>1× Moderate	4× Possibly<br>1× Related
Dizziness	3 (12.0%)		4 (16.7%)		3 (12.0%)		8 (30.8%)		5 (20.0%)	
	3× Mild	3× Possibly	4× Mild	4× Possibly	3× Mild	1× Not Related<br>1× Possibly<br>1× Related	8× Mild	1× Not Related<br>4× Possibly<br>3× Related	4× Mild<br>1× Moderate	1× Not Related<br>1× Unlikely<br>2× Possibly<br>1× Related
Hypoaesthesia	2 (8.0%)		1 (4.2%)		2 (8.0%)		1 (3.8%)		2 (8.0%)	
	1× Mild<br>1× Moderate	2× Related	1× Mild	1× Possibly	1× Mild<br>1× Moderate	2× Related	1× Mild	1× Possibly	1× Mild<br>1× Moderate	2× Not Related
Dysgeusia	2 (8.0%)		0		2 (8.0%)		3 (11.5%)		0	

	2× Mild	2× Related			2× Mild	1× Not Related<br>1× Possibly	3× Mild	1× Not Related<br>1× Possibly<br>1× Related		
Burning sensation	0		1 (4.2%)		1 (4.0%)		2 (7.7%)		2 (8.0%)	
			1× Mild	1× Possibly	1× Mild	1× Possibly	1× Mild<br>1× Severe	1× Possibly<br>1× Related	2× Mild	2× Possibly
Hypoaesthesia facial	1 (4.0%)		1 (4.2%)		2 (8.0%)		0		1 (4.0%)	
	1× Mild	1× Related	1× Mild	1× Possibly	2× Mild	1× Possibly<br>1× Related			1× Mild	1× Possibly
Oral dysaesthesia	1 (4.0%)		1 (4.2%)		1 (4.0%)		2 (7.7%)		0	
	1× Mild	1× Related	1× Mild	1× Related	1× Mild	1× Related	2× Mild	2× Not Related		
Somnolence	1 (4.0%)		0		0		1 (3.8%)		2 (8.0%)	
	1× Mild	3× Possibly					1× Mild	1× Related	2× Mild	2× Possibly
Hyperaesthesia	1 (4.0%)		0		0		1 (3.8%)		1 (4.0%)	
	1× Moderate	1× Related					1× Mild	1× Possibly	1× Mild	1× Related
Neuropathy peripheral	0		1 (4.2%)		0		2 (7.7%)		0	
			1× Mild	1× Related			2× Moderate	1× Not Related<br>1× Related		
Pharyngeal hypoaesthesia	1 (4.0%)		0		1 (4.0%)		1 (3.8%)		0	
	1× Mild	1× Related			1× Mild	1× Related	1× Mild	1× Not Related		
Hypoaesthesia teeth	0		0		0		2 (7.7%)		0	
							2× Mild	2× Possibly		
Neuralgia	1 (4.0%)		0		0		1 (3.8%)		0	
	1× Mild	1× Not Related					1× Moderate	1× Related		
Peripheral sensory neuropathy	0		0		1 (4.0%)		1 (3.8%)		0	
					1× Moderate	1× Not Related	1× Moderate	1× Related		
Sensory disturbance	0		1 (4.2%)		0		0		1 (4.0%)	
			1× Mild	1× Unlikely					1× Mild	1× Possibly

Amnesia	0	0	1 (4.0%)<br>1× Mild 1× Not Related	0	0
Ataxia	0	0	0	1 (3.8%)<br>1× Moderate 1× Related	0
Dysaesthesia	0	0	1 (4.0%)<br>1× Mild 1× Related	0	0
Dyscalculia	0	0	1 (4.0%)<br>1× Mild 1× Not Related	0	0
Head discomfort	0	0	0	1 (3.8%)<br>1× Mild 1× Related	0
Hypersomnia	0	0	0	1 (3.8%)<br>1× Moderate 1× Unlikely	0
Hyporeflexia	0	0	0	0	1 (4.0%)<br>1× Mild 1× Possibly
Lethargy	0	0	0	0	1 (4.0%)<br>1× Moderate 1× Possibly
Motor dysfunction	0	1 (4.2%)<br>1× Mild 1× Possibly	0	0	0
Post-traumatic headache	0	1 (4.2%)<br>1× Mild 1× Not Related	0	0	0
**General disorders and administration site conditions**					
Fatigue	4 (16.0%)<br>4× Mild 1× Unlikely<br>1× Possibly<br>2× Related	5 (20.8%)<br>4× Mild 1× Not Related<br>1× Moderate 3× Possibly<br>1× Related	5 (20.0%)<br>5× Mild 3× Not Related<br>1× Unlikely<br>1× Possibly	3 (11.5%)<br>3× Mild 1× Not Related<br>1× Unlikely<br>1× Possibly	4 (16.0%)<br>3× Mild 2× Unlikely<br>1× Moderate 2× Possibly
Feeling hot	1 (4.0%)<br>1× Mild 1× Related	1 (4.2%)<br>1× Mild 1× Possibly	1 (4.0%)<br>1× Mild 1× Possibly	2 (7.7%)<br>2× Mild 1× Related<br>1× Possibly	2 (8.0%)<br>2× Mild 2× Possibly
Pain	1 (4.0%)<br>1× Moderate 1× Related	2 (8.3%)<br>2× Mild 1× Unlikely<br>1× Possibly	1 (4.0%)<br>1× Moderate 1× Not Related	1 (3.8%)<br>1× Severe 1× Related	1 (4.0%)<br>1× Mild 1× Not Related
Feeling abnormal	0	1 (4.2%)<br>1× Mild 1× Possibly	2 (8.0%)<br>2× Mild 2× Related	1 (3.8%)<br>1× Mild 1× Possibly	1 (4.0%)<br>1× Mild 1× Possibly
Injection site pain	2 (8.0%)	2 (8.3%)	0	1 (3.8%)	0

	2× Mild	2× Possibly	2× Mild	2× Possibly			1× Mild	1× Possibly		
Feeling drunk	1 (4.0%)		1 (4.2%)		0		1 (3.8%)		1 (4.0%)	
	1× Mild	1× Possibly	1× Mild	1× Related			1× Mild	1× Related	1× Mild	1× Possibly
Oedema peripheral	1 (4.0%)		1 (4.2%)		0		1 (3.8%)		1 (4.0%)	
	1× Mild	1× Not Related	1× Mild	1× Possibly			1× Mild	1× Not Related	1× Mild	1× Possibly
Chest discomfort	0		1 (4.2%)		1 (4.0%)		0		0	
			1× Mild	1× Related	1× Mild	1× Related				
Influenza like illness	0		0		0		2 (7.7%)		0	
							1× Mild	1× Not Related		
							1× Moderate	1× Possibly		
Facial pain	0		0		0		0		1 (4.0%)	
									1× Mild	1× Possibly
Feeling cold	1 (4.0%)		0		0		0		0	
	1× Mild	1× Possibly								
Gait disturbance	1 (4.0%)		0		0		0		0	
	1× Mild	1× Possibly								
Injection site erythema	1 (4.0%)		0		0		0		0	
	1× Mild	1× Possibly								
Local swelling	0		0		0		1 (3.8%)		0	
							1× Mild	1× Not Related		
Therapeutic response unexpected	0		0		0		1 (3.8%)		0	
							1× Mild	1× Possibly		
**Gastrointestinal disorders**										
Nausea	1 (4.0%)		5 (20.8%)		1 (4.0%)		6 (23.1%)		6 (24.0%)	
	1× Moderate	1× Possibly	3× Mild	1× Not Related	1× Mild	1× Possibly	4× Mild	1× Unlikely	5× Mild	1× Not Related
			2× Moderate	3× Possibly			2× Moderate	3× Possibly	1× Moderate	1× Unlikely
				1× Related				2× Related		3× Possibly
										1× Related
Diarrhoea	2 (8.0%)		1 (4.2%)		0		2 (7.7%)		0	
	2× Mild	1× Unlikely	1× Mild	1× Not Related			1× Mild	1× Unlikely		
		1× Possibly					1× Moderate	1× Related		

Abdominal discomfort	1 (4.0%)<br>1× Mild 1× Not Related	1 (4.2%)<br>1× Moderate 1× Not Related	0	1 (3.8%)<br>1× Mild 1× Possibly	1 (4.0%)<br>1× Mild 1× Possibly
Constipation	0	1 (4.2%)<br>1× Moderate 1× Unlikely	1 (4.0%)<br>1× Mild 1× Not Related	1 (3.8%)<br>1× Mild 1× Related	0
Abdominal distension	1 (4.0%)<br>1× Moderate 1× Not Related	0	0	0	1 (4.0%)<br>1× Mild 1× Unlikely
Abdominal pain	1 (4.0%)<br>1× Mild 1× Unlikely	0	0	1 (3.8%)<br>1× Mild 1× Not Related	0
Abdominal pain upper	1 (4.0%)<br>1× Mild 1× Possibly	0	0	0	1 (4.0%)<br>1× Moderate 1× Not Related
Lip swelling	0	0	0	1 (3.8%)<br>1× Mild 1× Not Related	1 (4.0%)<br>1× Mild 1× Possibly
Tongue disorder	1 (4.0%)<br>1× Mild 1× Related	0	1 (4.0%)<br>1× Mild 1× Not Related	0	0
Vomiting	0	0	0	1 (3.8%)<br>1× Moderate 1× Related	1 (4.0%)<br>1× Mild 1× Unlikely
Abdominal rigidity	1 (4.0%)<br>1× Mild 1× Possibly	0	0	0	0
Aphthous stomatitis	0	0	1 (4.0%)<br>1× Mild 1× Not Related	0	0
Dry mouth	0	0	0	1 (3.8%)<br>1× Mild 1× Possibly	0
Flatulence	1 (4.0%)<br>1× Mild 1× Possibly	0	0	0	0
Lip dry	0	1 (4.2%)<br>1× Mild 1× Related	0	0	0
Lip oedema	0	0	0	0	1 (4.0%)<br>1× Mild 1× Possibly
Salivary hypersecretion	0	0	1 (4.0%)<br>1× Mild 1× Not Related	0	0
**Musculoskeletal and connective tissue disorders**					

Pain in extremity	1 (4.0%)		5 (20.8%)		4 (16.0%)		3 (11.5%)		2 (8.0%)	
	1× Mild	1× Not Related	4× Mild<br>1× Moderate	1× Unlikely<br>3× Possibly<br>1× Related	1× Mild<br>3× Moderate	2× Not Related<br>1× Possibly<br>1× Related	2× Mild<br>1× Severe	1× Not Related<br>1× Possibly<br>1× Related	1× Mild<br>1× Moderate	1× Possibly<br>1× Related
Back pain	2 (8.0%)		1 (4.2%)		1 (4.0%)		3 (11.5%)		3 (12.0%)	
	2× Moderate	2× Not Related	1× Mild	1× Not Related	1× Moderate	1× Not Related	2× Mild<br>1× Moderate	2× Unlikely<br>1× Not Related	2× Mild<br>1× Moderate	1× Unlikely<br>2× Not Related
Arthralgia	1 (4.0%)		1 (4.2%)		1 (4.0%)		2 (7.7%)		2 (8.0%)	
	1× Mild	1× Not Related	1× Moderate	1× Not Related	1× Moderate	1× Not Related	2× Mild	1× Not Related<br>1× Related	2× Mild	1× Unlikely<br>1× Related
Muscle spasms	0		1 (4.2%)		0		2 (7.7%)		1 (4.0%)	
			1× Mild	1× Not Related			1× Mild<br>1× Moderate	1× Not Related<br>1× Related	1× Mild	1× Related
Musculoskeletal stiffness	0		1 (4.2%)		0		1 (3.8%)		1 (4.0%)	
			1× Mild	1× Possibly			1× Mild	1× Possibly	1× Mild	1× Possibly
Myalgia	1 (4.0%)		0		0		0		1 (4.0%)	
	1× Moderate	1× Not Related							1× Mild	1× Possibly
Neck pain	0		0		0		0		2 (8.0%)	
									2× Mild	1× not Related<br>1× Possibly
Sensation of heaviness	0		0		0		1 (3.8%)		1 (4.0%)	
							1× Mild	1× Unlikely	1× Mild	1× Possibly
Flank pain	0		0		1 (4.0%)		0		0	
					1× Mild	1× Not Related				
Joint stiffness	0		1 (4.2%)		0		0		0	
			1× Mild	1× Possibly						
Limb discomfort	0		1 (4.2%)		0		0		0	
			1× Mild	1× Possibly						
Muscle tightness	0		0		0		0		1 (4.0%)	
									1× Mild	1× Possibly
Muscle twitching	0		0		0		0		1 (4.0%)	
									1× Mild	1× Related

Musculoskeletal chest pain	0	1 (4.2%)<br>1× Mild 1× Not Related	0	0	0
Musculoskeletal pain	0	0	0	0	1 (4.0%)<br>1× Mild 1× Not Related
Osteoarthritis	1 (4.0%)<br>1× Mild 1× Not Related	0	0	0	0
Pain in jaw	0	0	0	1 (3.8%)<br>1× Moderate 1× Not Related	0
**Respiratory, thoracic and mediastinal disorders**					
Cough	1 (4.0%)<br>1× Mild 1× Not Related	0	0	1 (3.8%)<br>1× Moderate 1× Unlikely	1 (4.0%)<br>1× Moderate 1× Not Related
Oropharyngeal pain	1 (4.0%)<br>1× Mild 1× Not Related	0	1 (4.0%)<br>1× Mild 1× Not Related	0	1 (4.0%)<br>1× Moderate 1× Not Related
Rhinitis allergic	0	1 (4.2%)<br>1× Moderate 1× Not Related	1 (4.0%)<br>1× Mild 1× Not Related	1 (3.8%)<br>1× Moderate 1× Not Related	0
Dyspnoea	0	0	0	1 (3.8%)<br>1× Mild 1× Unlikely	1 (4.0%)<br>1× Mild 1× Not Related
Nasal congestion	0	0	0	1 (3.8%)<br>1× Moderate 1× Not Related	1 (4.0%)<br>1× Mild 1× Not Related
Bronchial hyperreactivity	0	0	0	1 (3.8%)<br>1× Mild 1× Not Related	0
Chronic obstructive pulmonary disease	0	0	0	1 (3.8%)<br>1× Moderate 1× Unlikely	0
Oropharyngeal discomfort	0	0	0	0	1 (4.0%)<br>1× Mild 1× Related
Pharyngeal oedema	0	0	1 (4.0%)<br>1× Mild 1× Possibly	0	0
Rhinorrhoea	0	0	1 (4.0%)<br>1× Mild 1× Not Related	0	0
Sneezing	0	1 (4.2%)	0	0	0

		1× Mild 1× Not Related			
Throat tightness	0	1 (4.2%)<br>1× Mild 1× Related	0	0	0
**Infections and infestations**					
Urinary tract infection	2 (8.0%)<br>2× Moderate 2× Not Related	0	1 (4.0%)<br>1× Mild 1× Not Related	0	1 (4.0%)<br>1× Moderate 1× Not Related
Nasopharyngitis	1 (4.0%)<br>1× Mild 1× Not Related	0	1 (4.0%)<br>1× Mild 1× Not Related	0	0
Upper respiratory tract infection	0	1 (4.2%)<br>1× Mild 1× Not Related	0	0	1 (4.0%)<br>1× Moderate 1× Not Related
Cystitis	0	1 (4.2%)<br>1× Mild 1× Not Related	0	0	0
Influenza	1 (4.0%)<br>1× Moderate 1× Not Related	0	0	0	0
Oral herpes	0	0	1 (4.0%)<br>1× Moderate 1× Not Related	0	0
Pharyngitis	0	1 (4.2%)<br>1× Mild 1× Not Related	0	0	0
Pneumonia	1 (4.0%)<br>1× Moderate 1× Not Related	0	0	0	0
Viral upper respiratory tract infection	0	0	0	1 (3.8%)<br>1× Severe 1× Unlikely	0
**Vascular disorders**					
Hot flush	1 (4.0%)<br>1× Mild 1× Related	1 (4.2%)<br>1× Mild 1× Related	0	2 (7.7%)<br>1× Mild 1× Not Related<br>1× Moderate 1× Related	1 (4.0%)<br>1× Mild 1× Possibly
Flushing	0	0	2 (8.0%)<br>2× Mild 2× Related	1 (3.8%)<br>1× Mild 1× Related	1 (4.0%)<br>1× Mild 1× Possibly
Hypertension	0	0	0	2 (7.7%)<br>1× Mild 2× Possibly<br>1× Severe	0

Intra-abdominal haematoma	0	1 (4.2%)<br>1× Mild 1× Possibly	0	0	0
Peripheral coldness	0	0	0	1 (3.8%)<br>1× Mild 1× Possibly	0
**Skin and subcutaneous tissue disorders**					
Pruritus	3 (12.0%)<br>3× Mild 1× Possibly<br>2× Related	1 (4.2%)<br>1× Mild 1× Possibly	0	0	0
Hyperhidrosis	0	1 (4.2%)	0	0	1 (4.0%)<br>1× Mild 1× Unlikely
Blister	0	0	0	1 (3.8%)<br>1× Moderate 1× Not Related	0
Dry skin	0	0	1 (4.0%)<br>1× Mild 1× Not Related	0	0
Erythema	0	0	0	0	1 (4.0%)<br>1× Mild 1× Possibly
Rash	0	1 (4.2%)<br>1× Mild 1× Possibly	0	0	0
Scar	0	0	0	0	1 (4.0%)<br>1× Mild 1× Not Related
Skin discoloration	1 (4.0%)<br>1× Mild 1× Possibly	0	0	0	0
**Injury, poisoning and procedural complications**					
Fall	0	2 (8.3%)<br>2× Mild 2× Not Related	0	0	1 (4.0%)<br>1× Mild 1× Not Related
Contusion	0	0	1 (4.0%)<br>1× Mild 1× Not Related	0	0
Joint sprain	0	0	0	1 (3.8%)<br>1× Mild 1× Not Related	0
Meniscus lesion	1 (4.0%)<br>1× Mild 1× Not Related	0	0	0	0
Pelvic fracture	1 (4.0%)<br>1× Moderate 1× Not Related	0	0	0	0

Post procedural discomfort	0	0	0	1 (3.8%)<br>1× Mild 1× Not Related	0
Procedural pain	0	0	0	0	1 (4.0%)<br>1× Mild 1× Not Related
**Ear and labyrinth disorders**					
Ear pain	0	0	1 (4.0%)<br>1× Mild 1× Not Related	1 (3.8%)<br>1× Mild 1× Not Related	0
Tinnitus	0	0	0	1 (3.8%)<br>1× Mild 1× Not Related	1 (4.0%)<br>1× Mild 1× Related
Vertigo	0	1 (4.2%)<br>1× Moderate 1× Related	0	1 (3.8%)<br>1× Moderate 1× Possibly	0
Ear pruritus	1 (4.0%)<br>1× Mild 1× Possibly	0	0	0	0
**Psychiatric disorders**					
Thinking abnormal	1 (4.0%)<br>1× Mild 1× Possibly	1 (4.2%)<br>1× Mild 1× Possibly	0	0	1 (4.0%)<br>1× Mild 1× Possibly
An×iety	0	0	0	0	1 (4.0%)<br>1× Moderate 1× Possibly
Hypervigilance	1 (4.0%)<br>1× Moderate 1× Not Related	0	0	0	0
Insomnia	0	1 (4.2%)<br>1× Moderate 1× Not Related	0	0	0
Nightmare	1 (4.0%)<br>1× Moderate 1× Related	0	0	0	0
**Eye disorders**					
Eye pain	1 (4.0%)<br>1× Mild 1× Possibly	1 (4.2%)<br>1× Mild 1× Unlikely	0	0	0
Vision blurred	0	0	0	1 (3.8%)<br>1× Mild 1× Related	1 (4.0%)<br>1× Mild 1× Not Related
Abnormal sensation in eye	1 (4.0%)<br>1× Mild 1× Possibly	0	0	0	0
Chalazion	0	0	1 (4.0%)<br>1× Moderate 1× Not Related	0	0

Diplopia	0	0	0	1 (3.8%)<br>1× Mild 1× Related	0
**Investigations**					
Body temperature increased	0	0	1 (4.0%)<br>1× Mild 1× Not Related	0	1 (4.0%)<br>1× Mild 1× Possibly
Blood glucose increased	0	0	1 (4.0%)<br>1× Mild 1× Not Related	0	0
Blood pressure increased	0	0	1 (4.0%)<br>1× Mild 1× Possibly	0	0
Electrocardiogram QT prolonged	0	0	1 (4.0%)<br>1× Mild 1× Possibly	0	0
Weight increased	0	0	0	0	1 (4.0%)<br>1× Mild 1× Unlikely
**Renal and urinary disorders**					
Dysuria	0	1 (4.2%)<br>1× Moderate 1× Not Related	0	0	0
Pollakiuria	0	1 (4.2%)<br>1× Moderate 1× Not Related	0	0	0
Renal pain	0	0	1 (4.0%)<br>1× Mild 1× Not Related	0	0
**Blood and lymphatic system disorders**					
Lymphadenopathy	0	0	1 (4.0%)<br>1× Mild 1× Not Related	0	1 (4.0%)<br>1× Mild 1× Not Related
**Cardiac disorders**					
Palpitations	1 (4.0%)<br>1× Mild 1× Not Related	0	0	0	0
Tachycardia	0	0	0	0	1 (4.0%)<br>1× Mild 1× Unlikely
**Neoplasms benign, malignant and unspecified (incl cysts and polyps)**					
Colon cancer metastatic	0	0	0	0	1 (4.0%)

					1× Moderate 1× Not Related
Metastatic carcinoma of the bladder	0	1 (4.2%)<br>1× Death 1× Not Related	0	0	0
Prostate cancer	0	1 (4.2%)<br>1× Death 1× Not Related	0	0	0
**Metabolism and nutrition disorders**					
Decreased appetite	0	1 (4.2%)<br>1× Moderate 1× Not Related	0	0	0

TTX: tetrodotoxin; QoL: quality of life; BID: twice daily; QD: once daily; *n*: number of subjects
